# A Click Approach to Novel D-Ring-Substituted 16α-Triazolylestrone Derivatives and Characterization of Their Antiproliferative Properties

**DOI:** 10.1371/journal.pone.0118104

**Published:** 2015-02-18

**Authors:** Judit Molnár, Éva Frank, Renáta Minorics, Zalán Kádár, Imre Ocsovszki, Bruno Schönecker, János Wölfling, István Zupkó

**Affiliations:** 1 Department of Pharmacodynamics and Biopharmacy, University of Szeged, Szeged, Hungary; 2 Department of Organic Chemistry, University of Szeged, Szeged, Hungary; 3 Department of Biochemistry, University of Szeged, Szeged, Hungary; 4 Institute of Organic Chemistry and Macromolecular Chemistry, Friedrich Schiller University Jena, Jena, Germany; Medical University of South Carolina, UNITED STATES

## Abstract

A simple and efficient synthesis of novel, D-ring substituted estrone derivatives containing a 16α-triazolyl moiety is described. Two epimeric azido alcohols (16α-azido-17α-hydroxy and 16α-azido-17β-hydroxy) of estra-1,3,5(10)-triene-3-methyl ether were prepared, followed by copper(I)-catalyzed azide-alkyne cycloaddition with various terminal alkynes. The steroidal triazoles were obtained in high yields and their activities against three human cancer cell lines (HeLa, MCF7 and A431) were screened. The most effective analogs were submitted to additional experiments in order to characterize their antiproliferative properties. As evidenced by flow cytometry, the selected steroids induced a disturbance in the HeLa cell cycle in a concentration- and exposure-dependent manner, through an increase of the hypodiploid population (subG1) and a cell cycle arrest in the G2/M phase. A noncancerous human fibroblast cell line (MRC5) was used to determine the selectivities of these compounds. Fluorescent microscopy after Hoechst 33258 - propidium iodide (HOPI) double staining revealed nuclear condensation and disturbed cell membrane integrity. The enhanced activities of caspase-3 and caspase-9 without activation of caspase-8 in the treated cells indicated the activation of the intrinsic pathway of apoptosis. The levels of cell cycle regulators (CDK1, cyclin B1/B2 and cdc25B) were decreased and the ratio Bax/Bcl-2 was increased 24 h after the treatment of HeLa cells (determined at an mRNA level by means of an RT-PCR technique). Under the same conditions, two agents elicited substantially increased degrees of phosphorylation of stathmin, as evidenced by Western blotting. The presented results demonstrate that these steroids can be regarded as appropriate structural scaffolds for the design and synthesis of further steroid analogs as innovative drug candidates with good efficacy.

## Introduction

Since cancerous disorders are the second leading cause of death worldwide, following cardiovascular diseases, improvement of their treatment is currently one of the greatest challenges. A survey of epidemiological data from 184 countries suggested that the global burden of cancer will increase to 23.6 million new cases each year by 2030, an increase of 68% compared with 2012 [1].

Steroids are a group of compounds that play versatile roles as anticancer agents. In hormone-dependent tumors such as breast, uterine, ovarian, prostate and endometrial cancers, the overexpression of steroid receptors is involved in enhanced cell proliferation. Different approaches have been devised to reduce the growth-stimulating hormonal response of cancer cells. These compounds are classified as steroidal antihormonal/antiproliferative anticancer agents. Additionally, a broad variety of steroidal molecules have either been isolated from natural sources or rationally designed and synthetized, and have been reported to exhibit efficacy against cancer cells through nonhormonal mechanisms. Cytotoxic steroids exert their actions on various molecular targets (e.g. microtubules or topoisomerase), usually leading to cell cycle blockade and apoptosis [2].

Since the discovery of Cu-catalyzed azide-alkyne 1,3-dipolar **c**ycloaddition (CuAAC) [3, 4], this technique has found numerous applications in a wide range of disciplines, including pharmaceutical sciences [5–8]. Certain advantageous properties, including versatility, regiospecificity, lack of byproducts and high conversions, have made click chemistry an ideal tool for the synthesis of compound libraries available for initial screening and for structure–activity profiling. Not surprisingly, a number of compounds containing a triazole moiety have been reported to exert biological activity, including antibacterial [9], antiallergic [10] and anti-HIV [11] effects. Introduction of a triazole ring at position 3 of the natural triterpene betulinic acid resulted in a set of compounds with considerable antiproliferative potency and proapoptotic capacity [12]. The introduction of a triazole moiety into the podophyllotoxin skeleton yielded conjugates with significant topoisomerase-II-inhibiting activity, and some of these new compounds proved more potent than the clinically used etoposide [13].

The synthesis of steroidal heterocycles has also attracted considerable interest in view of their valuable pharmacological activities [14, 15]. Steroidal azoles have been described as potent inhibitors of 17α-hydroxylase-C_17,20_-lyase (CYP17), which can block androgen synthesis at an early stage, and may therefore be of use in the treatment of prostatic carcinoma [16, 17]. Furthermore, some heterocyclic derivatives have been found to exert strong inhibitory effects on 5α-reductases [18].

Banday *et al*. recently reported the syntheses of some 21-triazoles of pregnenolone as potent anticancer agents through a click chemistry approach [19]. In this regard, we have demonstrated that a number of triazolyl androstanes can exert direct cytostatic effects on human cancer cell lines *in vitro* [20–22]. Although the introduction of substituted triazole rings at position 17 of the estrane skeleton has so far met with only limited success as concerns the antiproliferative activity [23], the synthetic modification of compounds in the estrone series still seems to provide excellent possibilities in the search for novel derivatives with noteworthy biological effects [24].

A triazole ring has been successfully utilized as a linker for the preparation of estradiol-containing agents based on anticancer natural products. The most active conjugate inhibited the growth of cancer cell lines at submicromolar concentrations, exerted disruption of the microtubule network, and disturbed the cell cycle distribution of MCF7 cells and the induction of apoptosis. These properties were explained by the downregulation of cyclin-dependent kinase 1 (CDK1) and the upregulation of crucial tumor suppressors (p21 and p53) [25].

Thus, we set out to devise an effective route for the preparation of estrone-derived triazoles containing the heteroring at position 16, via CuAAC. Although determination of the affinities for the hormonal receptor did not fall within the scope of the present study, the presence of a methoxy group instead of a hydroxy group on C-3 and the steric bulk caused by the incorporated heteroring on C-16 are considered to influence the ability of these molecules to bind to the hormone receptor, thereby preventing the development of a significant estrogenic effect. All of the prepared compounds were screened *in vitro* for their activities against three human cancer cell lines (HeLa, MCF7 and A431). The most effective four compounds were selected for additional experiments with the aim of characterizing their antiproliferative properties.

## Experimental

### General methods

Reagents and materials were obtained from commercial suppliers and were used without purification. All solvents were distilled immediately prior to use. Reactions were monitored by TLC on Kieselgel-G (Merck Si 254 F) layers (0.25 mm thick). Spots were detected by spraying with 5% phosphomolybdic acid in 50% aqueous phosphoric acid and observed by illumination at 254 and 365 nm. Flash chromatography: Merck silica gel 60, 40–63 μm. Melting points (mps) were determined on a Kofler block and are uncorrected. Elemental analysis data were obtained with a Perkin Elmer CHN analyzer model 2400. IR spectra were recorded on a Perkin-Elmer FT-IR Spectrum 100. NMR spectra were recorded at room temperature with a Bruker DRX 500 instrument at 500 MHz (^1^H NMR) or 125 MHz (13C NMR). Chemical shifts are reported in ppm (*δ* scale), and coupling constants (*J*) in Hz. For the determination of multiplicities, the *J*-MOD pulse sequence was used. Automated flow injection analyses were performed by using an HPLC/MSD system. The system comprised an Agilent 1100 micro vacuum degasser, a quaternary pump, a micro-well plate autoinjector and a 1946A MSD equipped with an electrospray ion source (ESI) operated in positive ion mode. The ESI parameters were: nebulizing gas N_2_, at 35 psi; drying gas N_2_, at 350°C and 12 L/min; capillary voltage 3000 V; and fragmentor voltage 70 V. The MSD was operated in scan mode with the mass range *m/z* 60−620. Samples (0.2 μL) were injected with an automated needle wash directly into the solvent flow (0.3 mL/min) of MeCN/H_2_O 70:30 (v/v) supplemented with 0.1% formic acid. The system was controlled by Agilent LC/MSD Chemstation software.

### Chemical syntheses

**16α-Azido-3-methoxyestra-1,3,5(10)-trien-17α-ol (2) and 16α-azido-3-methoxyestra-1,3,5(10)-trien-17β-ol (3)**. Compound **1** (16α-azido-3-methoxyestra-1,3,5(10)-trien-17-one; 9.76 g, 30 mmol) was dissolved in a mixture of CH_2_Cl_2_ (60 mL) and MeOH (240 mL), the solution was cooled in an ice-bath to 15 ^ο^C, and KBH_4_ (6.5 g, 120.5 mmol) was added in small portions. The mixture was allowed to stand for 20 min, then poured into water (500 mL), neutralized with concentrated AcOH and extracted with CH_2_Cl_2_ (3 × 50 mL). The combined organic layers were washed with water, dried over Na_2_SO_4_ and evaporated *in vacuo*. The residual crude product was chromatographed on silica gel with CH_2_Cl_2_ to obtain **2** as a white solid (4.1 g, 42%), mp 95–98°C [97–99°C [26]], ^1^H NMR (CDCl_3_): *δ*_H_ 0.76 (s, 3H, 18-H_3_), 2.27 (m, 1H, 9-H), 2.86 (m, 2H, 6-H_2_), 3.75 (d, 1H, *J* = 5.0 Hz, 17-H), 3.79 (s, 3H, OMe), 4.20 (m, 1H, 16-H), 6.63 (d, 1H, *J* = 2.0 Hz, 4-H), 6.72 (dd, 1H, *J* = 8.5 Hz, *J* = 2.0 Hz, 2-H), 7.21 (d, 1H, *J* = 8.5 Hz, 1-H); 13C NMR (CDCl_3_):*δ*_C_ 17.1 (C-18), 25.7, 28.0, 29.7, 31.0, 31.1, 38.7 (C-8), 43.4 (C-9), 45.6 (C-13), 46.8 (C-14), 55.2 (OMe), 63.4 (C-16), 79.3 (C-17), 111.5 (C-2), 113.8 (C-4), 126.2 (C-1), 132.4 (C-10), 137.8 (C-5), 157.5 (C-3); Anal. calcd. for C_19_H_25_N_3_O_2_: C, 69.70; H, 7.70. Found: C, 69.56; H, 7.76.

Continued elution with CH_2_Cl_2_ resulted in **3** as a white solid (5.3 g, 54%), mp 114–116°C [112–114°C [26]], ^1^H NMR (CDCl_3_): *δ*_H_ 0.83 (s, 3H, 18-H_3_), 2.24 (m, 1H, 9-H), 2.86 (m, 2H, 6-H_2_), 3.63 (d, 1H, *J* = 6.5 Hz, 17-H), 3.78 (s, 3H, OMe), 3.82 (m, 1H, 16-H), 6.64 (d, 1H, *J* = 2.0 Hz, 4-H), 6.73 (dd, 1H, *J* = 8.5 Hz, *J* = 2.0 Hz, 2-H), 7.20 (d, 1H, *J* = 8.5 Hz, 1-H); 13C NMR (CDCl_3_): *δ*_C_ 11.9 (C-18), 25.8, 27.1, 29.6, 30.5, 36.3, 38.2, 43.7, 43.8 (C-13), 48.2, 55.2 (OMe), 67.0 (C-16), 87.2 (C-17), 111.5 (C-2), 113.8 (C-4), 126.2 (C-1), 132.1 (C-10), 137.7 (C-5), 157.5 (C-3); Anal. calcd. for C_19_H_25_N_3_O_2_: C, 69.70; H, 7.70. Found: C, 69.82; H, 7.55.

**General procedure for the preparation of 16α-triazolyl-3-methoxyestra-1,3,5(10)-trien-17-ols (4a–l and 5a–l)**. Compound **2** or **3** (327 mg, 1.00 mmol) was dissolved in dry CH_2_Cl_2_ (10 mL), and a solution of CuSO_4·_5H_2_O (12.5 mg, 5 mol%) and sodium ascorbate (30 mg, 15 mol%) in water (10 mL) was poured into the organic phase. The appropriate terminal alkyne (1.00 mmol) was added to the reaction mixture, which was then stirred overnight at ambient temperature. After the consumption of the starting material (TLC monitoring), the two-phase solution was diluted with water (30 mL) and extracted with CH_2_Cl_2_ (2 x 30 mL). The combined organic layers were washed with water, dried over Na_2_SO_4_ and evaporated *in vacuo*. The resulting crude product was purified by flash chromatography with EtOAc/CH_2_Cl_2_ (5:95).

**16α-(4-Phenyl-1H-1,2,3-triazol-1-yl)-3-methoxyestra-1,3,5(10)-trien-17α-ol (4a)**. According to section 2.2.2, azidoalcohol **2** and phenylacetylene (0.11 mL) were added to the mixture. Product: **4a** (385 mg), mp 222–224°C, ^1^H NMR (CDCl_3_): *δ*_H_ 0.97 (s, 3H, 18-H_3_), 1.46–1.58 (overlapping m, 2H), 1.61 (m, 1H), 1.73 (m, 1H), 1.87 (m, 1H), 2.04 (m, 1H), 2.16 (m, 1H), 2.22–2.31 (overlapping m, 2H), 2.38–2.46 (overlapping m, 2H), 2.88 (m, 2H, 6-H_2_), 3.79 (s, 3H, OMe), 4.22 (d, 1H, *J* = 5.0 Hz, 17-H), 5.46 (m, 1H, 16-H), 6.66 (d, 1H, *J* = 2.0 Hz, 4-H), 6.75 (dd, 1H, *J* = 8.5 Hz, *J* = 2.0 Hz, 2-H), 7.21–7.27 (overlapping m, 4H, 1-H, 3″-H, 4″-H and 5″-H), 7.52 (d, 2H, *J* = 8.0 Hz, 2″- and 6″-H), 7.81 (s, 1H, 5′-H); 13C NMR (CDCl_3_): *δ*_C_ 17.4 (C-18), 25.8 (CH_2_), 28.0 (CH_2_), 29.8 (CH_2_), 31.2 (CH_2_), 32.6 (CH_2_), 38.8 (CH), 43.4 (CH), 45.9 (C-13), 47.2 (CH), 55.2 (OMe), 62.7 (C-16), 79.4 (C-17), 111.6 (C-2), 113.8 (C-4), 119.9 (C-5′), 125.2 (2C, C-2″ and C-6″), 126.3 (C-1), 127.6 (C-4″), 128.6 (2C, C-3″ and C-5″), 130.1 (C-1″), 132.4 (C-10), 137.7 (C-5), 146.9 (C-4′), 157.5 (C-3); Anal. calcd. for C_27_H_31_N_3_O_2_: C, 75.49; H, 7.27. Found: C, 75.60; H, 7.33.

**16α-[4-(3-Tolyl)-1H-1,2,3-triazol-1-yl]-3-methoxyestra-1,3,5(10)-trien-17α-ol (4b)**. According to section 2.2.2, azidoalcohol **2** and 3-tolylacetylene (0.13 mL) were added to the mixture. Product: **4b** (402 mg), mp 185–187°C, ^1^H NMR (CDCl_3_): *δ*_H_ 0.97 (s, 3H, 18-H_3_), 1.44–1.54 (overlapping m, 2H), 1.60 (m, 1H), 1.73 (m, 1H), 1.87 (m, 1H), 2.03 (m, 1H), 2.16 (m, 1H), 2.26 (m, 2H), 2.27 (s, 3H, 3″-CH_3_), 2.37–2.45 (overlapping m, 2H), 2.88 (m, 2H, 6-H_2_), 3.79 (s, 3H, OMe), 4.21 (d, 1H, *J* = 5.0 Hz, 17-H), 5.44 (m, 1H, 16-H), 6.66 (d, 1H, *J* = 2.0 Hz, 4-H), 6.75 (dd, 1H, *J* = 8.5 Hz, *J* = 2.0 Hz, 2-H), 7.03 (d, 1H, *J* = 7.5 Hz, 4″-H), 7.19 (t, 1H, *J* = 7.5 Hz, 5″-H), 7.25 (d, 1H, *J* = 8.5 Hz, 1-H), 7.40 (bs, 1H, 2″-H), 7.52 (d, 1H, *J* = 7.5 Hz, 6″-H), 7.79 (s, 1H, 5′-H); 13C NMR (CDCl_3_): *δ*_C_ 17.3 (C-18), 21.3 (3″-CH_3_), 25.8 (CH_2_), 28.0 (CH_2_), 29.8 (CH_2_), 31.2 (CH_2_), 32.6 (CH_2_), 38.7 (CH), 43.4 (CH), 45.8 (C-13), 47.2 (CH), 55.2 (OMe), 62.7 (C-16), 79.4 (C-17), 111.5 (C-2), 113.8 (C-4), 119.9 (C-5′), 122.3 (C-6″), 125.9 (C-2″), 126.3 (C-1), 128.4 and 128.5 (C-4″ and C-5″), 130.0 (C-1″), 132.4 (C-10), 137.7 (C-5), 138.0 (C-3″), 147.0 (C-4′), 157.5 (C-3); Anal. Calcd. For C_28_H_33_N_3_O_2_: C, 75.81; H, 7.50. Found: C, 75.67; H, 7.46.

**16α-[4-(4-Tolyl)-1H-1,2,3-triazol-1-yl]-3-methoxyestra-1,3,5(10)-trien-17α-ol (4c)**. According to section 2.2.2, azidoalcohol **2** and 4-tolylacetylene (0.13 mL) were added to the mixture. Product: **4c** (410 mg), mp 173–175 ^ο^C, ^1^H NMR (CDCl_3_): *δ*_H_ 0.95 (s, 3H, 18-H_3_), 1.44–1.53 (overlapping m, 2H), 1.59 (m, 1H), 1.72 (m, 1H), 1.86 (m, 1H), 2.03 (m, 1H), 2.13 (m, 1H), 2.20–2.29 (overlapping m, 2H), 2.33 (s, 3H, 4″-CH_3_), 2.36–2.44 (overlapping m, 2H), 2.87 (m, 2H, 6-H_2_), 3.78 (s, 3H, OMe), 4.19 (d, 1H, *J* = 5.0 Hz, 17-H), 5.43 (m, 1H, 16-H), 6.65 (d, 1H, *J* = 2.0 Hz, 4-H), 6.74 (dd, 1H, *J* = 8.5 Hz, *J* = 2.0 Hz, 2-H), 7.05 (d, 2H, *J* = 8.0 Hz, 3″- and 5″-H), 7.24 (d, 1H, *J* = 8.5 Hz, 1-H), 7.41 (d, 2H, *J* = 8.0 Hz, 2″-H and 6″-H), 7.76 (s, 1H, 5′-H); 13C NMR (CDCl_3_): *δ*_C_ 17.3 (C-18), 21.2 (4″-CH_3_), 25.8 (CH_2_), 28.0 (CH_2_), 29.8 (CH_2_), 31.2 (CH_2_), 32.5 (CH_2_), 38.7 (CH), 43.4 (CH), 45.8 (C-13), 47.2 (CH), 55.2 (OMe), 62.7 (C-16), 79.5 (C-17), 111.5 (C-2), 113.8 (C-4), 119.6 (C-5′), 125.1 (2C, C-3″ and C-2″), 126.3 (C-1), 127.3 (C-1″), 129.2 (2C, C-2″ and C-6″), 132.4 (C-10), 137.4 (C-4″), 137.8 (C-5), 146.9 (C-4′), 157.5 (C-3); Anal. Calcd. For C_28_H_33_N_3_O_2_: C, 75.81; H, 7.50. Found: C, 75.99; H, 7.42.

**16α-[4-(4-Methoxyphenyl)-1H-1,2,3-triazol-1-yl]-3-methoxyestra-1,3,5(10)-trien-17α-ol (4d)**. According to section 2.2.2, azidoalcohol **2** and 4-methoxyphenylacetylene (132 mg) were added to the mixture. Product: **4d** (370 mg), mp 132–134^ο^C; ^1^H NMR (CDCl_3_): *δ*_H_ 0.96 (s, 3H, 18-H_3_), 1.45–1.54 (overlapping m, 2H), 1.60 (m, 1H), 1.72 (m, 1H), 1.87 (m, 1H), 2.03 (m, 1H), 2.14 (m, 1H), 2.21–2.30 (overlapping m, 2H), 2.37–2.45 (overlapping m, 2H), 2.88 (m, 2H, 6-H_2_), 3.79 (s, 3H, 3-OMe), 3.82 (s, 3H, 4″-OMe), 4.21 (d, 1H, *J* = 5.0 Hz, 17-H), 5.44 (m, 1H, 16-H), 6.65 (d, 1H, *J* = 2.0 Hz, 4-H), 6.74–6.78 (overlapping m, 3H, 2-H, 3″-H and 5″-H), 7.27 (d, 1H, *J* = 8.5 Hz, 1-H), 7.43 (d, 2H, *J* = 8.5 Hz, 2″-H and 6″-H), 7.71 (s, 1H, 5′-H); 13C NMR (CDCl_3_): *δ*_C_ 17.3 (C-18), 25.8 (CH_2_), 28.0 (CH_2_), 29.8 (CH_2_), 31.2 (CH_2_), 32.5 (CH_2_), 38.7 (CH), 43.4 (CH), 45.8 (C-13), 47.2 (CH), 55.2 (2C, 3-OMe, 4″-OMe), 62.7 (C-16), 79.4 (C-17), 111.5 (C-2), 113.7 (C-4), 113.9 (2C, C-3″ and C-5″), 119.1 (C-5′), 122.9 (C-1″), 126.3 (C-1), 126.5 (2C, C-2″ and C-6″), 132.4 (C-10), 137.7 (C-5), 146.6 (C-4′), 157.4 (C-3), 159.1 (C-4″); Anal. calcd. for C_28_H_33_N_3_O_3_: C, 73.18; H, 7.24. Found: C, 73.29; H, 7.16.

**16α-[4-(2-Methoxyphenyl)-1H-1,2,3-triazol-1-yl]-3-methoxyestra-1,3,5(10)-trien-17α-ol (4e)**. According to section 2.2.2, azidoalcohol **2** and 2-methoxyphenylacetylene (0.13 mL) were added to the mixture. Product: **4e** (365 mg), mp 252–254 ^ο^C, ^1^H NMR (CDCl_3_): *δ*_H_ 0.96 (s, 3H, 18-H_3_), 1.45–1.64 (overlapping m, 3H), 1.71 (m, 1H), 1.89 (m, 1H), 2.10 (m, 1H), 2.19–2.33 (overlapping m, 3H), 2.37–2.44 (overlapping m, 2H), 2.89 (m, 2H, 6-H_2_), 3.79 (s, 3H, OMe), 3.86 (s, 3H, 2″-OCH_3_), 4.17 (d, 1H, *J* = 5.0 Hz, 17-H), 5.42 (m, 1H, 16-H), 6.66 (d, 1H, *J* = 2.0 Hz, 4-H), 6.74 (dd, 1H, *J* = 8.5 Hz, *J* = 2.0 Hz, 2-H), 6.83 (d, 1H, *J* = 8.0 Hz, 3″-H), 7.02 (t, 1H, *J* = 7.5 Hz, 5″-H), 7.22–7.26 (overlapping m, 2H, 1-H and 4″-H), 8.09 (s, 1H, 5′-H), 8.16 (d, 1H, *J* = 7.5 Hz, 6″-H) 13C NMR (CDCl_3_): *δ*_C_ 17.4 (C-18), 25.8 (CH_2_), 28.1 (CH_2_), 29.8 (CH_2_), 31.1 (CH_2_), 32.2 (CH_2_), 38.8 (CH), 43.4 (CH), 45.9 (C-13), 47.2 (CH), 55.2 and 55.3 (3-OMe, 2″-OMe), 62.5 (C-16), 80.0 (C-17), 110.5 (C-3″), 111.5 (C-2), 113.8 (C-4), 119.1 (C-1″), 120.8 (C-5″), 123.4 (C-5′), 126.3 (C-1), 127.3 and 128.6 (C-4″ and C-6″), 132.4 (C-10), 137.8 (C-5), 142.7 (C-4′), 155.4 (C-2″), 157.5 (C-3); Anal. calcd. for C_28_H_33_N_3_O_3_: C, 73.18; H, 7.24. Found: C, 73.22; H, 7.27.

**16α-[4-(4-tert-Butylphenyl)-1H-1,2,3-triazol-1-yl]-3-methoxyestra-1,3,5(10)-trien-17α-ol (4f)**. According to section 2.2.2, azidoalcohol **2** and 4-*tert*-butylphenylacetylene (0.18 mL) were added to the mixture. Product: 4f (435 mg), mp 181–183 ^ο^C, ^1^H NMR (CDCl_3_): *δ*_H_ 0.96 (s, 3H, 18-H_3_), 1.34 (s, 9H, 3 *t*Bu-CH_3_), 1.46–1.55 (overlapping m, 2H), 1.61 (m, 1H), 1.73 (m, 1H), 1.87 (m, 1H), 2.04 (m, 1H), 2.16 (m, 1H), 2.21–2.32 (overlapping m, 2H), 2.38–2.45 (overlapping m, 2H), 2.88 (m, 2H, 6-H_2_), 3.79 (s, 3H, OMe), 4.21 (d, 1H, *J* = 5.0 Hz, 17-H), 5.46 (m, 1H, 16-H), 6.66 (d, 1H, *J* = 2.0 Hz, 4-H), 6.75 (dd, 1H, *J* = 8.5 Hz, *J* = 2.0 Hz, 2-H), 7.26–7.30 (overlapping m, 3H, 1-H, 3″-H and 5″-H), 7.46 (d, 2H, *J* = 8 Hz, 2″-H and 6″-H), 7.76 (s, 1H, 5′-H); 13C NMR (CDCl_3_): *δ*_C_ 17.4 (C-18), 25.9 (CH_2_), 28.0 (CH_2_), 29.8 (CH_2_), 31.2 (CH_2_), 31.3 (3C, 3 *t*Bu-CH_3_), 32.5 (CH_2_), 34.5 (*t*Bu-C), 38.8 (CH), 43.4 (CH), 45.8 (C-13), 47.2 (CH), 55.2 (OMe), 62.7 (C-16), 79.4 (C-17), 111.6 (C-2), 113.8 (C-4), 119.6 (C-5′), 125.0 (2C), 125.4 (2C), 126.3 (C-1), 127.4 (C-1″), 132.4 (C-10), 137.8 (C-5), 146.9 (C-4′), 150.6 (C-4″), 157.5 (C-3); Anal. calcd. for C_31_H_39_N_3_O_2_: C, 76.67; H, 8.09. Found: C, 76.85; H, 8.14.

**16α-[4-(4-Ethylphenyl)-1H-1,2,3-triazol-1-yl]-3-methoxyestra-1,3,5(10)-trien-17α-ol (4g)**. According to section 2.2.2, azidoalcohol **2** and 4-ethylphenylacetylene (0.14 mL) were added to the mixture. Product: **4g** (405 mg), mp 113–115 ^ο^C, ^1^H NMR (CDCl_3_): *δ*_H_ 0.97 (s, 3H, 18-H_3_), 1.26 (t, 3H, *J* = 7.5 Hz, CH_2_ CH_3_), 1.48–1.55 (overlapping m, 2H), 1.61 (m, 1H), 1.74 (m, 1H), 1.87 (m, 1H), 2.04 (m, 1H), 2.17 (m, 1H), 2.21–2.32 (overlapping m, 2H), 2.38–2.46 (overlapping m, 2H), 2.63 (q, 2H, *J* = 7.5 Hz, CH_2_CH_3_), 2.89 (m, 2H, 6-H_2_), 3.80 (s, 3H, OMe), 4.23 (d, 1H, *J* = 5.0 Hz, 17-H), 5.46 (m, 1H, 16-H), 6.66 (d, 1H, *J* = 2.0 Hz, 4-H), 6.76 (dd, 1H, *J* = 8.5 Hz, *J* = 2.0 Hz, 2-H), 7.06 (d, 2H, *J* = 8.0 Hz, 3″-H and 5″-H), 7.27 (d, 1H, *J* = 8.5 Hz, 1-H), 7.41 (d, 2H, *J* = 8 Hz, 2″-H and 6″-H), 7.76 (s, 1H, 5′-H); 13C NMR (CDCl_3_): *δ*_C_ 15.5 (CH_2_ CH_3_), 17.4 (C-18), 25.8 (CH_2_), 28.0 (CH_2_), 28.6 (CH_2_CH_3_), 29.8 (CH_2_), 31.2 (CH_2_), 32.5 (CH_2_), 38.7 (CH), 43.4 (CH), 45.8 (C-13), 47.2 (CH), 55.2 (OMe), 62.7 (C-16), 79.4 (C-17), 111.5 (C-2), 113.8 (C-4), 119.5 (C-5′), 125.1 (2C, C-2″ and C-6″), 126.3 (C-1), 127.5 (C-1″), 128.0 (2C, C-3″ and C-5″), 132.4 (C-10), 137.8 (C-5), 143.6 (C-4″), 146.9 (C-4′), 157.4 (C-3); Anal. calcd. for C_29_H_35_N_3_O_2_: C, 76.12; H, 7.71. Found: C, 76.26; H, 7.58.

**16α-[4-(4-Propylphenyl)-1H-1,2,3-triazol-1-yl]-3-methoxyestra-1,3,5(10)-trien-17α-ol (4h)**. According to section 2.2.2, azidoalcohol **2** and 4-*n*-propylphenylacetylene (0.16 mL) were added to the mixture. Product: **4h** (435 mg), mp 105–107 ^ο^C, ^1^H NMR (CDCl_3_): *δ*_H_ 0.96–0.98 (overlapping multiplets, 6H, 18-H_3_ and CH_3_CH_2_CH_2_), 1.65 (m, 2H, CH_3_ CH_2_CH_2_), 1.48–1.54 (overlapping m, 2H), 1.59–1.68 (overlapping m, 3H, CH_3_ CH_2_CH_2_ and 1H), 1.73 (m, 1H), 1.87 (m, 1H), 2.03 (m, 1H), 2.17 (m, 1H), 2.20–2.31 (overlapping m, 2H), 2.38–2.45 (overlapping m, 2H), 2.56 (t, 2H, *J* = 7.5 Hz, CH_3_CH_2_ CH_2_), 2.89 (m, 2H, 6-H_2_), 3.79 (s, 3H, OMe), 4.22 (d, 1H, *J* = 5.0 Hz, 17-H), 5.45 (m, 1H, 16-H), 6.66 (d, 1H, *J* = 2.0 Hz, 4-H), 6.76 (dd, 1H, *J* = 8.5 Hz, *J* = 2.0 Hz, 2-H), 7.05 (d, 2H, *J* = 8.0 Hz, 3″-H and 5″-H), 7.27 (d, 1H, *J* = 8.5 Hz, 1-H), 7.41 (d, 2H, *J* = 8.0 Hz, 2″-H and 6″-H), 7.76 (s, 1H, 5′-H); 13C NMR (CDCl_3_): *δ*_C_ 13.8 (CH_3_CH_2_CH_2_), 17.3 (C-18), 24.4 (CH_3_ CH_2_CH_2_), 25.8 (CH_2_), 28.0 (CH_2_), 29.8 (CH_2_), 31.2 (CH_2_), 32.5 (CH_2_), 37.7 (CH_3_CH_2_ CH_2_), 38.7 (CH), 43.4 (CH), 45.8 (C-13), 47.2 (CH), 55.2 (OMe), 62.7 (C-16), 79.4 (C-17), 111.5 (C-2), 113.8 (C-4), 119.6 (C-5′), 125.0 (2C, C-2″ and C-6″), 126.3 (C-1), 127.5 (C-1″), 128.6 (2C, C-3″ and C-5″), 132.4 (C-10), 137.7 (C-5), 142.1 (C-4″), 146.9 (C-4′), 157.4 (C-3); Anal. calcd. for C_30_H_37_N_3_O_2_: C, 76.40; H, 7.91. Found: C, 76.52; H, 7.86.

**16α-[4-(3-Aminophenyl)-1H-1,2,3-triazol-1-yl]-3-methoxyestra-1,3,5(10)-trien-17α-ol (4i)**. According to section 2.2.2, azidoalcohol **2** and 3-aminophenylacetylene (0.11 mL) were added to the mixture. Product: **4i** (334 mg), mp 124–126 ^ο^C, ^1^H NMR (CDCl_3_): *δ*_H_ 0.94 (s, 3H, 18-H_3_), 1.46–1.52 (overlapping m, 2H), 1.58 (m, 1H), 1.70 (m, 1H), 1.86 (m, 1H), 2.04 (m, 1H), 2.11 (m, 1H), 2.20–2.28 (overlapping m, 2H), 2.34–2.43 (overlapping m, 2H), 2.88 (m, 2H, 6-H_2_), 3.79 (s, 3H, OMe), 4.16 (d, 1H, *J* = 5.0 Hz, 17-H), 5.43 (m, 1H, 16-H), 6.57 (d, 1H, *J* = 8.0 Hz, 4″-H), 6.65 (d, 1H, *J* = 2.0 Hz, 4-H), 6.74 (dd, 1H, *J* = 8.5 Hz, *J* = 2.0 Hz, 2-H), 6.93 (s, 1H, 2″-H), 6.99 (d, 1H, *J* = 8.0 Hz, 6″-H), 7.09 (t, 1H, *J* = 8.0 Hz, 5″-H), 7.25 (d, 1H, *J* = 8.5 Hz, 1-H), 7.77 (s, 1H, 5′-H); 13C NMR (CDCl_3_): *δ*_C_ 17.0 (C-18), 25.5 (CH_2_), 27.7 (CH_2_), 29.4 (CH_2_), 30.9 (CH_2_), 32.2 (CH_2_), 38.4 (CH), 43.0 (CH), 45.5 (C-13), 46.9 (CH), 54.8 (OMe), 62.3 (C-16), 79.1 (C-17), 111.2 (C-2), 111.8 (C-4″), 113.4 (C-4), 114.3 (C-2″), 115.6 (C-6″), 119.8 (C-5′), 126.0 (C-1), 129.1 (C-5″), 130.9 (C-1″), 132.0 (C-10), 137.4 (C-5), 146.3 (C-3″), 146.7 (C-4′), 157.1 (C-3); Anal. calcd. for C_27_H_32_N_4_O_2_: C, 72.94; H, 7.26. Found: C, 73.05; H, 7.14.

**16α-(4-Cyclopropyl-1H-1,2,3-triazol-1-yl)-3-methoxyestra-1,3,5(10)-trien-17α-ol (4j)**. According to section 2.2.2, azidoalcohol **2** and cyclopropylacetylene (0.09 mL) were added to the mixture. Product: **4j** (342 mg), mp 221–223 ^ο^C, ^1^H NMR (CDCl_3_): *δ*_H_ 0.82 (m, 2H), 0.91–0.94 (overlapping m, 5H, 18-H_3_ and 2H), 1.45–1.52 (overlapping m, 2H), 1.57 (m, 1H), 1.67 (m, 1H), 1.86 (m, 1H), 1.91 (m, 1H), 2.08 (m, 1H), 2.13–2.22 (overlapping m, 2H), 2.34 (m, 1H), 2.41 (m, 2H), 2.88 (m, 2H, 6-H_2_), 3.78 (s, 3H, OMe), 4.06 (d, 1H, *J* = 5.0 Hz, 17-H), 5.33 (m, 1H, 16-H), 6.64 (d, 1H, *J* = 2.0 Hz, 4-H), 6.74 (dd, 1H, *J* = 8.5 Hz, *J* = 2.0 Hz, 2-H), 7.24 (d, 1H, *J* = 8.5 Hz, 1-H), 7.35 (s, 1H, 5′-H); 13C NMR (CDCl_3_): *δ*_C_ 6.7 (CH_2_), 7.5 (CH_2_), 7.6 (C), 17.3 (C-18), 25.8 (CH_2_), 28.0 (CH_2_), 29.8 (CH_2_), 31.2 (CH_2_), 32.3 (CH_2_), 38.7 (CH), 43.3 (CH), 45.8 (C-13), 47.2 (CH), 55.2 (OMe), 62.4 (C-16), 79.5 (C-17), 111.5 (C-2), 113.8 (C-4), 120.1 (C-5′), 126.3 (C-1), 132.4 (C-10), 137.7 (C-5), 149.2 (C-4′), 157.5 (C-3); Anal. calcd. for C_24_H_31_N_3_O_2_: C, 73.25; H, 7.94. Found: C, 73.33; H, 7.90.

**16α-(4-Cyclopentyl-1H-1,2,3-triazol-1-yl)-3-methoxyestra-1,3,5(10)-trien-17α-ol (4k)**. According to section 2.2.2, azidoalcohol **2** and cyclopentylacetylene (0.12 mL) were added to the mixture. Product: **4k** (334 mg), mp 156–158 ^ο^C, ^1^H NMR (CDCl_3_): *δ*_H_ 0.93 (s, 3H, 18-H_3_), 1.45–1.53 (overlapping m, 2H), 1.57–1.74 (overlapping m, 8H), 1.87 (m, 1H), 2.00–2.05 (overlapping m, 4H), 2.15–2.22 (overlapping m, 2H), 2.34–2.43 (overlapping m, 2H), 2.88 (m, 2H, 6-H_2_), 3.03 (m, 1H, 1″-H), 3.79 (s, 3H, OMe), 4.13 (d, 1H, *J* = 5.0 Hz, 17-H), 5.44 (m, 1H, 16-H), 6.65 (d, 1H, *J* = 2.0 Hz, 4-H), 6.74 (dd, 1H, *J* = 8.5 Hz, *J* = 2.0 Hz, 2-H), 7.24 (d, 1H, *J* = 8.5 Hz, 1-H), 7.38 (s, 1H, 5′-H); 13C NMR (CDCl_3_): *δ*_C_ 17.3 (C-18), 25.0 (CH_2_), 25.1 (CH_2_), 25.8 (CH_2_), 28.0 (CH_2_), 29.8 (CH_2_), 31.2 (CH_2_), 32.3 (CH_2_), 32.8 (CH_2_), 33.0 (CH_2_), 36.7 (CH), 38.7 (CH), 43.3 (CH), 45.8 (C-13), 47.2 (CH), 55.2 (OMe), 62.3 (C-16), 79.5 (C-17), 111.5 (C-2), 113.8 (C-4), 120.0 (C-5′), 126.3 (C-1), 132.4 (C-10), 137.7 (C-5), 151.8 (C-4′), 157.4 (C-3); Anal. calcd. for C_26_H_35_N_3_O_2_: C, 74.07; H, 8.37. Found: C, 74.13; H, 8.29.

**16α-(4-Cyclohexyl-1H-1,2,3-triazol-1-yl)-3-methoxyestra-1,3,5(10)-trien-17α-ol (4l)**. According to section 2.2.2, azidoalcohol **2** and cyclohexylacetylene (0.13 mL) were added to the mixture. Product: **4l** (380 mg, 87%), mp 159–161 ^ο^C, ^1^H NMR (CDCl_3_): *δ*_H_ 0.93 (s, 3H, 18-H_3_), 1.22–1.41 (overlapping m, 4H), 1.44–1.62 (overlapping m, 6H), 1.70 (m, 2H), 1.78 (m, 1H), 1.87 (m, 1H), 2.02–2.10 (overlapping m, 4H), 2.15–2.25 (overlapping m, 2H), 2.34–2.43 (overlapping m, 2H), 2.62 (m, 1H, 1″-H), 2.88 (m, 2H, 6-H_2_), 3.79 (s, 3H, OMe), 4.13 (d, 1H, *J* = 5.0 Hz, 17-H), 5.37 (m, 1H, 16-H), 6.65 (d, 1H, *J* = 2.0 Hz, 4-H), 6.74 (dd, 1H, *J* = 8.5 Hz, *J* = 2.0 Hz, 2-H), 7.24 (d, 1H, *J* = 8.5 Hz, 1-H), 7.36 (s, 1H, 5′-H); 13C NMR (CDCl_3_): *δ*_C_ 17.3 (C-18), 25.8 (CH_2_), 26.0 (CH_2_), 26.1 (CH_2_), 28.0 (CH_2_), 29.8 (CH_2_), 31.2 (CH_2_), 32.3 (CH_2_), 32.6 (CH_2_), 33.0 (CH_2_), 35.1 (CH), 38.7 (CH), 43.3 (CH), 45.8 (C-13), 47.2 (CH), 55.2 (OMe), 62.3 (C-16), 79.5 (C-17), 111.5 (C-2), 113.8 (C-4), 119.8 (C-5′), 126.3 (C-1), 132.4 (C-10), 137.7 (C-5), 152.7 (C-4′), 157.4 (C-3); Anal. calcd. for C_27_H_37_N_3_O_2_: C, 74.45; H, 8.56. Found: C, 74.61; H, 8.52.

**16α-(4-Phenyl-1H-1,2,3-triazol-1-yl)-3-methoxyestra-1,3,5(10)-trien-17β-ol (5a)**. According to section 2.2.2, azidoalcohol **3** and phenylacetylene (0.11 mL) were added to the mixture. Product: **5a** (352 mg), mp 275–277 ^ο^C, ^1^H NMR (DMSO-*d*_6_): *δ*_H_ 0.85 (s, 3H, 18-H_3_), 1.30–1.44 (overlapping m, 4H), 1.79–1.87 (overlapping m, 3H), 1.95 (m, 1H), 2.13 (m, 1H), 2.25 (m, 1H), 2.34 (m, 1H), 2.80 (m, 2H, 6-H_2_), 3.70 (s, 3H, OMe), 3.93 (t, 1H, *J* = 6.0 Hz, 17-H), 4.80 (m, 1H, 16-H), 5.36 (d, 1H, *J* = 5.3 Hz, OH), 6.62 (d, 1H, *J* = 2.0 Hz, 4-H), 6.70 (dd, 1H, *J* = 8.6 Hz, *J* = 2.0 Hz, 2-H), 7.20 (d, 1H, *J* = 8.6 Hz, 1-H), 7.33 (t, 1H, *J* = 7.4 Hz, 4″-H), 7.45 (t, 2H, *J* = 7.7 Hz, 3″-H and 5″-H), 7.87 (m, 2H, J = 7.7 Hz, 2″-H and 6″-H), 8.70 (s, 1H, 5′-H); 13C NMR (DMSO-*d*_6_): *δ*_C_ 11.6 (C-18), 25.6, 26.6, 29.1, 31.6, 35.9, 38.0, 43.3, 43.5 (C-13), 47.6, 54.8 (OMe), 65.9 (C-16), 86.1 (C-17), 111.5 (C-2), 113.4 (C-4), 120.5 (C-5′), 125.0 (2C), 126.1 (C-1), 127.6 (C-4″), 128.8 (2C), 130.9 (C-1″), 131.8 (C-10), 137.3 (C-5), 146.3 (C-4′), 157.0 (C-3); Anal. calcd. for C_27_H_31_N_3_O_2_: C, 75.49; H, 7.27. Found: C, 75.60; H, 7.23.

**16α-[4-(3-Tolyl)-1H-1,2,3-triazol-1-yl]-3-methoxyestra-1,3,5(10)-trien-17β-ol (5b)**. According to section 2.2.2, azidoalcohol **3** and 3-tolylacetylene (0.13 mL) were added to the mixture. Product: **5b** (395 mg), mp 225–228 ^ο^C, ^1^H NMR (CDCl_3_): *δ*_H_ 0.97 (s, 3H, 18-H_3_), 1.43–1.50 (overlapping m, 2H), 1.55 (m, 1H), 1.82–1.89 (overlapping m, 2H), 2.03 (m, 1H), 2.18 (m, 1H), 2.26–2.32 (overlapping m, 2H), 2.36 (m, 1H), 2.39 (s, 3H, 3″-CH_3_), 2.37–2.45 (overlapping m, 2H), 2.87 (m, 2H, 6-H_2_), 3.79 (s, 3H, OMe), 4.18 (d, 1H, *J* = 7.0 Hz, 17-H), 4.74 (m, 1H, 16-H), 6.64 (d, 1H, *J* = 2.0 Hz, 4-H), 6.73 (dd, 1H, *J* = 8.5 Hz, *J* = 2.0 Hz, 2-H), 7.15 (d, 1H, *J* = 7.5 Hz, 4″-H), 7.21 (d, 1H, *J* = 8.5 Hz, 1-H), 7.30 (t, 1H, *J* = 7.5 Hz, 5″-H), 7.56 (d, 1H, *J* = 7.5 Hz, 6″-H), 7.60 (br s, 1H, 2″-H), 7.75 (s, 1H, 5′-H); Anal. calcd. for C_28_H_33_N_3_O_2_: C, 75.81; H, 7.50. Found: C, 75.92; H, 7.61.

**16α-[4-(4-Tolyl)-1H-1,2,3-triazol-1-yl]-3-methoxyestra-1,3,5(10)-trien-17β-ol (5c)**. According to section 2.2.2, azidoalcohol **3** and 4-tolylacetylene (0.13 mL) were added to the mixture. Product: **5c** (403 mg), mp 271–273 ^ο^C, ^1^H NMR (CDCl_3_): *δ*_H_ 0.97 (s, 3H, 18-H_3_), 1.44–1.52 (overlapping m, 2H), 1.55 (m, 1H), 1.82–1.89 (overlapping m, 2H), 2.02 (m, 1H), 2.19 (m, 1H), 2.29–2.35 (overlapping m, 4H), 2.36 (m, 1H), 2.39 (s, 3H, 4″-CH_3_), 2.88 (m, 2H, 6-H_2_), 3.78 (s, 3H, OMe), 4.18 (d, 1H, *J* = 7.0 Hz, 17-H), 4.75 (m, 1H, 16-H), 6.64 (d, 1H, *J* = 2.0 Hz, 4-H), 6.72 (dd, 1H, *J* = 8.5 Hz, *J* = 2.0 Hz, 2-H), 7.20–7.24 (overlapping multiplets, 3H, 1-H, 3″-H and 5″-H), 7.71 (d, 2H, *J* = 8 Hz, 2″-H and 6″-H), 7.78 (s, 1H, 5′-H); Anal. calcd. for C_28_H_33_N_3_O_2_: C, 75.81; H, 7.50. Found: C, 75.91; H, 7.39.

**16α-[4-(4-Methoxyphenyl)-1H-1,2,3-triazol-1-yl]-3-methoxyestra-1,3,5(10)-trien-17β-ol (5d)**. According to section 2.2.2, azidoalcohol **3** and 4-methoxyphenylacetylene (132 mg) were added to the mixture. Product: **5d** (405 mg), mp 259–262 ^ο^C, ^1^H NMR (CDCl_3_): *δ*_H_ 0.97 (s, 3H, 18-H_3_), 1.41–1.52 (overlapping m, 2H), 1.54 (m, 1H), 1.82–1.89 (overlapping m, 2H), 2.02 (m, 1H), 2.19 (m, 1H), 2.29–2.40 (overlapping m, 3H), 2.54 (m, 1H), 2.88 (m, 2H, 6-H_2_), 3.78 (s, 3H, OMe), 3.85 (s, 3H, 4″-OMe), 4.18 (d, 1H, *J* = 7.0 Hz, 17-H), 4.74 (m, 1H, 16-H), 6.64 (d, 1H, *J* = 2.0 Hz, 4-H), 6.72 (dd, 1H, *J* = 8.5 Hz, *J* = 2.0 Hz, 2-H), 6.95 (d, 2H, *J* = 8.0 Hz, 3″-H and 5″-H), 7.21 (d, 1H, *J* = 8.5 Hz, 1-H), 7.72 (d, 2H, *J* = 8.0 Hz, 2″-H and 6″-H), 7.75 (s, 1H, 5′-H); Anal. calcd. for C_28_H_33_N_3_O_3_: C, 73.18; H, 7.24. Found: C, 73.27; H, 7.18.

**16α-[4-(2-Methoxyphenyl)-1H-1,2,3-triazol-1-yl]-3-methoxyestra-1,3,5(10)-trien-17β-ol (5e)**. According to section 2.2.2, azidoalcohol **3** and 2-methoxyphenylacetylene (0.13 mL) were added to the mixture. Product: **5e** (400 mg), mp 242–244 ^ο^C, ^1^H NMR (CDCl_3_): *δ*_H_ 0.98 (s, 3H, 18-H_3_), 1.41–1.54 (overlapping m, 3H), 1.86–1.92 (overlapping m, 2H), 2.02 (m, 1H), 2.18 (m, 1H), 2.30–2.39 (overlapping m, 3H), 2.53 (m, 1H), 2.87 (m, 2H, 6-H_2_), 3.79 (s, 3H, OMe), 3.91 (s, 3H, 2″-OCH_3_), 4.24 (d, 1H, *J* = 7.0 Hz, 17-H), 4.74 (m, 1H, 16-H), 6.64 (d, 1H, *J* = 2.0 Hz, 4-H), 6.73 (dd, 1H, *J* = 8.5 Hz, *J* = 2.0 Hz, 2-H), 6.96 (d, 1H, *J* = 8.0 Hz, 3″-H), 7.08 (t, 1H, *J* = 8 Hz, 5″-H), 7.21 (d, 1H, *J* = 8.5 Hz, 1-H), 7.31 (t, 1H, *J* = 7.5 Hz, 4″-H), 8.07 (s, 1H, 5′-H), 8.33 (d, 1H, *J* = 8.0 Hz, 6″-H); Anal. calcd. for C_28_H_33_N_3_O_3_: C, 73.18; H, 7.24. Found: C, 73.22; H, 7.18.

**16α-[4-(4-tert-Butylphenyl)-1H-1,2,3-triazol-1-yl]-3-methoxyestra-1,3,5(10)-trien-17β-ol (5f)**. According to section 2.2.2, azidoalcohol **3** and 4-*tert*-butylphenylacetylene (0.18 mL) were added to the mixture. Product: **5f** (450 mg), mp 255–257 ^ο^C, ^1^H NMR (CDCl_3_): *δ*_H_ 0.97 (s, 3H, 18-H_3_), 1.35 (s, 9H, 3 *t*Bu-CH_3_), 1.41–1.55 (overlapping m, 3H), 1.83–1.89 (overlapping m, 2H), 2.02 (m, 1H), 2.18 (m, 1H), 2.30–2.39 (overlapping m, 3H), 2.76 (m, 1H), 2.88 (m, 2H, 6-H_2_), 3.78 (s, 3H, OMe), 4.18 (d, 1H, *J* = 7.0 Hz, 17-H), 4.74 (m, 1H, 16-H), 6.64 (d, 1H, *J* = 2.0 Hz, 4-H), 6.72 (dd, 1H, *J* = 8.5 Hz, *J* = 2.0 Hz, 2-H), 7.21 (d, 1H, *J* = 8.5 Hz, 1-H), 7.43 (d, 2H, *J* = 8.0 Hz, 3″-H and 5″-H), 7.71 (d, 2H, *J* = 8 Hz, 2″-H and 6″-H), 7.75 (s, 1H, 5’-H); ^13^C NMR (DMSO-*d*_6_): *δ* 11.6 (C-18), 25.6, 26.6, 29.1, 31.0 (3C, 3 × ^*t*^Bu-CH_3_), 31.6, 35.9, 38.0, 43.3, 43.5 (C-13), 47.5, 54.8 (OMe), 65.9 (C-16), 86.0 (C-17), 111.5 (C-2), 113.4 (C-4), 120.2 (C-5′), 124.8 (2C), 125.5 (2C), 126.1 (C-1), 128.1 (C-4″), 131.8 (C-1″), 137.3 (C-10), 146.3 (C-5), 150.1 (C-4′), 157.0 (C-3); Anal. calcd. for C_31_H_39_N_3_O_2_: C, 76.67; H, 8.09. Found: C, 76.75; H, 8.15.

**16α-[4-(4-Ethylphenyl)-1H-1,2,3-triazol-1-yl]-3-methoxyestra-1,3,5(10)-trien-17β-ol (5g)**. According to section 2.2.2, azidoalcohol **3** and 4-ethylphenylacetylene (0.14 mL) were added to the mixture. Product: **5g** (415 mg). mp 250–253 ^ο^C, ^1^H NMR (DMSO-*d*_6_): *δ*_H_ 0.85 (s, 3H, 18-H_3_), 1.20 (t, 3H, *J* = 7.5 Hz, CH_3_CH_2_CH_2_), 1.31–1.44 (overlapping m, 4H), 1.79–1.87 (overlapping m, 3H), 1.94 (m, 1H), 2.12 (m, 1H), 2.24 (m, 1H), 2.33 (m, 1H), 2.63 (q, 2H, *J* = 7.5 Hz, CH_3_ CH_2_), 2.78 (m, 2H, 6-H_2_), 3.69 (s, 3H, OMe), 3.94 (m, 1H, 17-H), 4.78 (m, 1H, 16-H), 5.34 (d, 1H, *J* = 5.1 Hz, OH), 6.62 (d, 1H, *J* = 1.8 Hz, 4-H), 6.69 (dd, 1H, *J* = 8.5 Hz, *J* = 1.8 Hz, 2-H), 7.19 (d, 1H, *J* = 8.5 Hz, 1-H), 7.28 (d, 2H, *J* = 7.9 Hz, 3″-H and 5″-H), 7.79 (d, 2H, *J* = 7.9 Hz, 2″-H and 6″-H), 8.63 (s, 1H, 5′-H); ^13^C NMR (DMSO-*d*_6_): *δ* 11.6 (C-18), 15.4 (CH_3_CH_2_), 25.6, 26.6, 27.8, 29.1, 31.6, 35.9, 38.0, 43.3, 43.5 (C-13), 47.5, 54.8 (OMe), 65.9 (C-16), 86.1 (C-17), 111.4 (C-2), 113.4 (C-4), 120.1 (C-5′), 125.0 (2C), 126.0 (C-1), 128.1 (2C), 128.4 (C-4″), 131.8 (C-1″), 137.3 (C-10), 143.2 (C-5), 146.4 (C-4′), 157.0 (C-3); Anal. calcd. for C_29_H_35_N_3_O_2_: C, 76.12; H, 7.71. Found: C, 76.23; H, 7.60.

**16α-[4-(4-Propylphenyl)-1H-1,2,3-triazol-1-yl]-3-methoxyestra-1,3,5(10)-trien-17β-ol (5h)**. According to section 2.2.2, azidoalcohol **3** and 4-*n*-propylphenylacetylene (0.16 mL) were added to the mixture. Product: **5h** (438 mg). mp 188–190 ^ο^C, ^1^H NMR (DMSO-*d*_6_): *δ*_H_ 0.85 (s, 3H, 18-H_3_), 0.90 (t, 3H, *J* = 7.3 Hz, CH_3_CH_2_CH_2_), 1.31–1.44 (overlapping m, 4H), 1.61 (m, 2H, CH_3_ CH_2_CH_2_), 1.78–1.87 (overlapping m, 3H), 1.95 (m, 1H), 2.12 (m, 1H), 2.24 (m, 1H), 2.33 (m, 1H), 2.57 (t, 2H, *J* = 7.5 Hz, CH_3_CH_2_ CH_2_), 2.78 (m, 2H, 6-H_2_), 3.69 (s, 3H, OMe), 3.94 (m, 1H, 17-H), 4.78 (m, 1H, 16-H), 5.34 (d, 1H, *J* = 5.2 Hz, OH), 6.62 (d, 1H, *J* = 1.7 Hz, 4-H), 6.69 (dd, 1H, *J* = 8.5 Hz, *J* = 1.7 Hz, 2-H), 7.19 (d, 1H, *J* = 8.5 Hz, 1-H), 7.26 (d, 2H, *J* = 7.9 Hz, 3″-H and 5″-H), 7.78 (d, 2H, *J* = 7.9 Hz, 2″-H and 6″-H), 8.63 (s, 1H, 5′-H); ^13^C NMR (DMSO-*d*_6_): *δ* 11.6 (C-18), 13.5 (CH_3_CH_2_CH_2_), 23.9, 25.6, 26.6, 29.1, 31.6, 35.9, 36.9, 38.0, 43.3, 43.5 (C-13), 47.5, 54.8 (OMe), 65.9 (C-16), 86.1 (C-17), 111.4 (C-2), 113.4 (C-4), 120.1 (C-5′), 124.9 (2C), 126.0 (C-1), 128.4 (C-4″), 128.7 (2C), 131.8 (C-1″), 137.3 (C-10), 141.6 (C-5), 146.4 (C-4′), 157.0 (C-3); Anal. calcd. for C_30_H_37_N_3_O_2_: C, 76.40; H, 7.91. Found: C, 76.52; H, 8.01.

**16α-[4-(3-Aminophenyl)-1H-1,2,3-triazol-1-yl]-3-methoxyestra-1,3,5(10)-trien-17β-ol (5i)**. According to section 2.2.2, azidoalcohol **3** and 3-aminophenylacetylene (0.11 mL) were added to the mixture. Product: **5i** (346 mg), oil, ^1^H NMR (CDCl_3_): *δ*_H_ 0.96 (s, 3H, 18-H_3_), 1.38–1.55 (overlapping m, 3H), 1.79–1.85 (overlapping m, 2H), 2.03 (m, 1H), 2.14 (m, 1H), 2.19–2.23 (overlapping m, 2H), 2.28–2.36 (overlapping m, 2H), 2.86 (m, 2H, 6-H_2_), 3.78 (s, 3H, OMe), 4.17 (d, 1H, *J* = 7.0 Hz, 17-H), 4.69 (m, 1H, 16-H), 6.63–6.65 (overlapping multiplets, 2H, 4- and 4″-H), 6.72 (dd, 1H, *J* = 8.5 Hz, *J* = 2.0 Hz, 2-H), 7.06–7.08 (overlapping multiplets, 2H, 2″- and 6″-H), 7.16–7.20 (overlapping multiplets, 2H, 1- and 5″-H), 7.64 (s, 1H, 5′-H); Anal. calcd. for C_27_H_32_N_4_O_2_: C, 72.94; H, 7.26. Found: C, 73.04; H, 7.32.

**16α-(4-Cyclopropyl-1H-1,2,3-triazol-1-yl)-3-methoxyestra-1,3,5(10)-trien-17β-ol (5j)**. According to section 2.2.2, azidoalcohol **3** and cyclopropylacetylene (0.085 mL) were added to the mixture. Product: **5j** (350 mg), mp 164–167 ^ο^C, ^1^H NMR (CDCl_3_): *δ*_H_ 0.82 (m, 2H), 0.92–0.95 (overlapping multiplets, 5H, 18-H_3_ and 2H), 1.38–1.58 (overlapping m, 3H), 1.76–1.85 (overlapping m, 2H), 2.00 (m, 1H), 2.13 (m, 1H), 2.18–2.23 (overlapping m, 2H), 2.28–2.37 (overlapping m, 2H), 2.86 (m, 2H, 6-H_2_), 3.78 (s, 3H, OMe), 4.11 (d, 1H, *J* = 6.5 Hz, 17-H), 4.65 (m, 1H, 16-H), 6.63 (d, 1H, *J* = 2.0 Hz, 4-H), 6.72 (dd, 1H, *J* = 8.5 Hz, *J* = 2.0 Hz, 2-H), 7.20 (d, 1H, *J* = 8.5 Hz, 1-H), 7.30 (s, 1H, 5′-H); Anal. calcd. for C_24_H_31_N_3_O_2_: C, 73.25; H, 7.94. Found: C, 73.34; H, 7.86.

**16α-(4-Cyclopentyl-1H-1,2,3-triazol-1-yl)-3-methoxyestra-1,3,5(10)-trien-17β-ol (5k)**. According to section 2.2.2, azidoalcohol **3** and cyclopentylacetylene (0.12 mL) were added to the mixture. Product: **5k** (354 mg), mp 166–168 ^ο^C, ^1^H NMR (CDCl_3_): *δ*_H_ 0.95 (s, 3H, 18-H_3_), 1.42–1.58 (overlapping m, 5H), 1.62–1.69 (overlapping m, 4H), 1.76–1.86 (overlapping m, 4H), 2.00 (m, 1H), 2.10 (m, 1H), 2.21–2.26 (overlapping m, 1H), 2.29–2.38 (overlapping m, 3H), 2.87 (m, 2H, 6-H_2_), 3.17 (m, 1H, 1″-H), 3.78 (s, 3H, OMe), 4.14 (d, 1H, *J* = 6.5 Hz, 17-H), 4.68 (m, 1H, 16-H), 6.63 (d, 1H, *J* = 2.0 Hz, 4-H), 6.72 (dd, 1H, *J* = 8.5 Hz, *J* = 2.0 Hz, 2-H), 7.21 (d, 1H, *J* = 8.5 Hz, 1-H), 7.33 (s, 1H, 5′-H); Anal. calcd. for C_26_H_35_N_3_O_2_: C, 74.07; H, 8.37. Found: C, 74.17; H, 8.45.

**16α-(4-Cyclohexyl-1H-1,2,3-triazol-1-yl)-3-methoxyestra-1,3,5(10)-trien-17β-ol (5l)**. According to section 2.2.2, azidoalcohol **3** and cyclohexylacetylene (0.13 mL) were added to the mixture. Product: **5l** (360 mg), mp 130–132 ^ο^C, ^1^H NMR (CDCl_3_): *δ*_H_ 0.95 (s, 3H, 18-H_3_), 1.42–1.56 (overlapping m, 6H), 1.72–1.86 (overlapping m, 6H), 1.98–2.37 (overlapping m, 8H), 2.49 (m, 1H), 2.73 (m, 1H, 1″-H), 2.87 (m, 2H, 6-H_2_), 3.78 (s, 3H, OMe), 4.14 (d, 1H, *J* = 6.5 Hz, 17-H), 4.68 (m, 1H, 16-H), 6.63 (d, 1H, *J* = 2.0 Hz, 4-H), 6.72 (dd, 1H, *J* = 8.5 Hz, *J* = 2.0 Hz, 2-H), 7.21 (d, 1H, *J* = 8.5 Hz, 1-H), 7.31 (s, 1H, 5′-H); Anal. calcd. for C_27_H_37_N_3_O_2_: C, 74.45; H, 8.56. Found: C, 74.67; H, 8.38.

### Cell culturing and determination of antiproliferative effects of the tested compounds

Human cell lines were purchased from ECACC (Salisbury, UK). HeLa (cervix adenocarcinoma), A431 (skin epidermoid carcinoma), MCF7 (breast adenocarcinoma) and noncancerous MRC-5 fetal lung fibroblast cells were cultivated in minimal essential medium supplemented with 10% fetal bovine serum, 1% non-essential amino acids and an antibiotic–antimycotic mixture. All media and supplements were obtained from PAA Laboratories GmbH, Pasching, Austria. Near-confluent cancer cells were seeded onto a 96-well microplate (5000/well) and attached to the bottom of the well overnight. On the second day, 200 μL of new medium containing the tested compound (at 10 or 30 μM) was added. After incubation for 72 h at 37°C in humidified air with 5% CO_2_, the living cells were assayed by the addition of 20 μL of 5 mg/mL MTT [3-(4,5-dimethylthiazol-2-yl)-2,5-diphenyltetrazolium bromide] solution. MTT was converted by intact mitochondrial reductase and precipitated as blue crystals during a 4-h contact period. The medium was then removed and the precipitated crystals were dissolved in 100 μL of DMSO during a 60-min period of shaking at 25°C. Finally, the reduced MTT was assayed at 545 nm, using a microplate reader; wells with untreated cells were utilized as controls [27]. For the most effective compounds, the assays were repeated with a set of dilutions, and sigmoidal dose–response curves were fitted to the measured data in order to determine the IC_50_ values by means of GraphPad Prism 4.0 (GraphPad Software; San Diego, CA, USA). All *in vitro* experiments were carried out on two microplates with at least five parallel wells. Cisplatin was used as positive control. Stock solutions of the tested substances (10 mM) were prepared with DMSO. The highest DMSO content of the medium (0.3%) did not have any substantial effect on the cell proliferation.

### Cell cycle analysis by flow cytometry

Cellular DNA content was determined by means of flow cytometric analysis, using a DNA- specific fluorescent dye, propidium iodide (PI). The cells were plated in a six-well plate and cultured for 24 h. The cultured cells were treated with various concentrations of the tested compounds for 24 h, after which the medium was removed, and the cells were washed with phosphate-buffered saline (PBS) and trypsinized. The harvested cells were suspended in medium and centrifuged at 1,700 rpm for 15 min at 4°C. The supernatant was then removed and the cells were resuspended in 1 mL of PBS. After the second centrifugation, 1 mL of -20°C 70% EtOH was added dropwise to the cell pellet. The cells were stored at -20°C until the day of DNA staining. On the day of DNA staining, the samples were washed with PBS and suspended in 1 mL of DNA staining buffer containing PI, ribonuclease-A, Triton-X and sodiumcitrate. After incubation for 1 h at room temperature, protected from light, the samples were analyzed by FACStar. For each experiment 20,000 events were counted, and the percentages of the cells in the different cell-cycle phases (subG1, G1, S and G2/M) were determined by means of winMDI 2.8 [28].

### Double staining with Hoechst 33258 and PI

Cells were seeded into a 96-well plate and incubated with various concentrations of the tested compounds for 24 h. The medium was then removed and 100 μL of medium with 10 μL of staining solution was added to the cells. The final concentrations of Hoechst 33258 and PI were 5 and 3 μg/mL, respectively. After incubation for 60 min at 37°C, the cells were examined on a Nikon Fluorescence Microscope equipped with a Digital Sight Camera System, including appropriate filters for Hoechst 33258 and PI [29, 30]

### Caspase-3 assay

Caspase-3 activity was determined by using a colorimetric assay kit (Sigma-Aldrich Ltd., Budapest, Hungary), Ac-DEVD-*p*NA serving as substrate. During the assay, the peptide substrate was cleaved by caspase-3, resulting in the release of *p*NA (*p*-nitroaniline), which was measured on a microplate reader at an absorbance wavelength of 405 nm. Caspase-3 activity was determined in the presence and absence of a selective inhibitor for caspase-3. HeLa cells were treated with the tested compounds at 3, 10 and 30 μM for 24 h; untreated cells were used as controls. Cells were scraped and incubated on ice with cell lysis buffer in proportion to the cell number for 15 min. The cell lysate was next centrifuged for 15 min at 17,000 g and the supernatant was collected and assayed by means of the microplate reader. Results were expressed in fold increase of caspase-3 activity compared with the control result [31].

### Caspase-8 assay

Caspase-8 activity was determined by using a colorimetric assay kit (Sigma-Aldrich Ltd., Budapest, Hungary), Ac-IETD-*p*NA serving as substrate. During the assay, the peptide substrate was cleaved by caspase-8, resulting in the release of *p*NA, which was measured on a microplate reader at an absorbance wavelength of 405 nm. All further conditions were identical with those of the caspase-3 assay.

### Caspase-9 assay

Caspase-9 activity was determined by using a colorimetric assay kit (Invitrogen; Carlsbad, CA, USA), with Ac-LEHD-*p*NA as substrate. During the assay, the peptide substrate was cleaved by caspase-9, resulting in the release of *p*NA, which was measured on a microplate reader at an absorbance wavelength of 405 nm. All further conditions were identical with those of the caspase-3 assay.

### Reverse transcription-polymerase chain reaction (RT-PCR) studies

The effects of the tested compounds on the mRNA expression pattern of the markers of apoptosis, such as Bax, Bcl-2, cyclin-dependent kinase 1 (CDK1), cdc25B, cyclin B1 and cyclin B2, which play a crucial role in the transition from the G2 to the M phase, were determined by RT-PCR in HeLa cells. After a 24-h incubation period, the total RNA was isolated from the cells (4×10^5^) through the use of TRIzol Reagent, in accordance with the instructions of the manufacturer (Csertex Ltd; Budapest, Hungary). The pellet was resuspended in 100 μL of DNase- and RNase-free distilled water. The RNA concentrations of the samples were determined from their absorbances at 260 nm. The RNA (0.5 μg) was mixed with DNase- and RNase-free distilled water and 20 μM oligodT (Invitrogen; Carlsbad, CA, USA), in a final reaction volume of 10 μL, and the mixture was incubated at 70°C for 5 min. After the mixture had been cooled to 4°C, 20 U of RNase inhibitor (Promega, Madison, WI, USA), 20 U of MMLV reverse transcriptase (Promega, Madison, USA), 200 μM dNTP (Sigma-Aldrich; Budapest, Hungary) in 50 mM Tris-HCl, pH 8.3, 75 mM KCl and 5 mM MgCl_2_ in a final reaction volume of 10 μL were added. The mixture was incubated at 37°C for 60 min. The PCR was carried out with 5 μL of cDNA, 12.5 μL of GoTaq Green Master Mix, 2 μL of 20 pM sense and the antisense primers of Bax, Bcl-2, CDK1, cdc25B, cyclin B1, cyclin B2 and 3.5 μL of DNase- and RNase-free distilled water. Human glyceraldehyde 3-phosphate dehydrogenase (hGAPDH) primers were used as internal control in all samples (S1 Table). The PCR was performed with an ESCO SWIFT MAXI thermal cycler (Esco Technologies; Philadelphia, PA, USA) and the products were separated on 2% agarose gels, stained with ethidium bromide and photographed under a UV transilluminator. Semiquantitative analysis was performed by densitometric scanning of the gel with a Kodak IMAGE STATION 2000R (Csertex; Budapest, Hungary).

### Western blotting studies

To investigate the actions of the most potent compounds on the functions of phosphorylated and total stathmin, protein expression was determined by using western blot analysis. HeLa cells were harvested in 60-mm dishes at a density of 2 x 10^5^ cells/mL and treated with the tested agents for 48 h. Whole-cell extracts were prepared by washing the cells with PBS and suspending them in lysis buffer (50 mM Tris, 5 mM EDTA, 150 mM NaCl, 1% NP-40, 0.5% deoxycholic acid, 1 mM sodium orthovanadate, 100 μg/mL PMSF and protease inhibitors) [32]. 10 μg of protein per well was subjected to electrophoresis on 4–12% NuPAGE Bis–Tris Gel in XCell SureLock Mini-Cell Units (Invitrogen, Carlsbad, CA, USA). Proteins were transferred from gels to nitrocellulose membranes, using the iBlot Gel Transfer System (Invitrogen, Carlsbad, CA, USA). Antibody binding was detected with the WesternBreeze Chemiluminescent Western blot immunodetection kit (Invitrogen, Carlsbad, CA, USA). The blots were incubated on a shaker with stathmin (Op18: rabbit polyclonal antibody raised against amino acids 1–149 representing full-length human protein), phosphorylated stathmin (p-Op18: rabbit polyclonal antibody raised against a short amino acid sequence containing phosphorylated Ser25 of human protein) and β-actin polyclonal antibody (Santa Cruz Biotechnology, Santa Cruz, CA, USA) 1:200 in the blocking buffer. Each sample was prepared in three parallels and the experiments were repeated twice. Semiquantitative analysis was performed by densitometric scanning of the blot with Kodak IMAGE STATION 2000R (Eastman Kodak Co., Rochester, NY, USA). All determined optical density values were normalized to the optical density value of β-actin. For statistical evaluation, data were analyzed by one-way ANOVA with the Neumann-Keuls post test, using GraphPad Prism version 4.0 for Windows (GraphPad Software, San Diego, CA, USA).

## Results

### Synthesis of 16α-triazolylestrone derivatives

Treatment of 16α-azido-3-methoxyestra-1,3,5(10)-trien-17-one (**1**) with KBH_4_ in MeOH/CH_2_Cl_2_ (4:1) resulted in two diastereomeric azidoalcohols. The mixture of epimers was separated by flash chromatography to furnish **2** (17α-OH) and **3** (17β-OH) in a ratio of ∼2:3. The similar reduction of **1** with LiBH_4_ in Et_2_O was reported earlier to result in the related 16,17-*cis* and -*trans* isomers (**2** and **3**) in a nearly 1:1 ratio [26].

**Fig 1:**
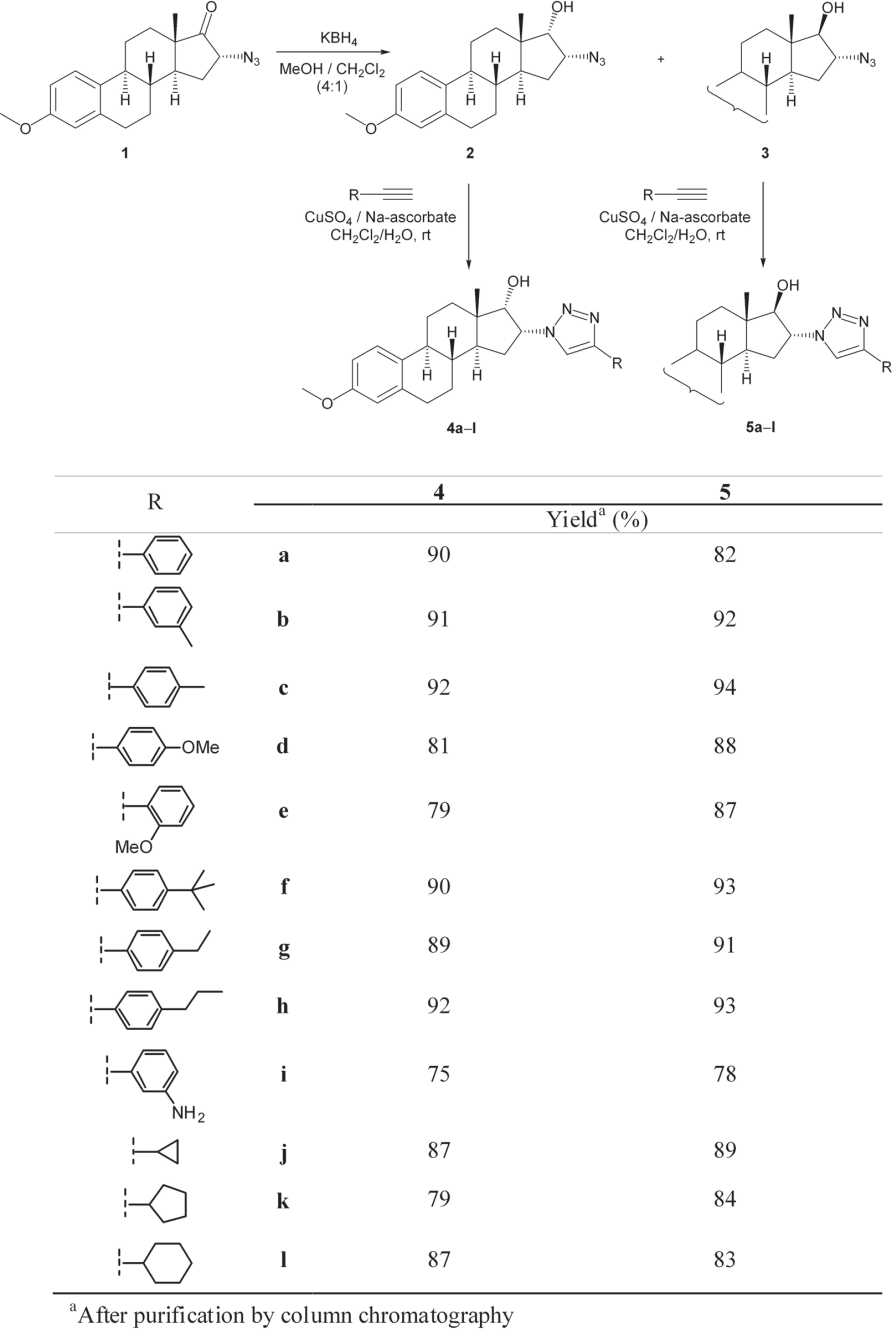
Synthesis of steroidal 16α-triazoles by CuAAc.

Several 16α-1,2,3-triazolyl derivatives (**4a**–**l** and **5a**–**l**) were next synthetized in good to excellent yields through the reactions of **2** or **3** with various terminal alkynes (Fig. 1). Since a Cu(I)-catalyzed process is approximately 10^7^ times faster than the uncatalyzed version, the intermolecular cycloaddition readily occurs at room temperature. As the application of Cu(I) salts in such reactions is known to require high temperature or at least the presence of an amine base additive (DIPEA or Et_3_N) for sufficient formation of the Cu-acetylide intermediate, and certain chelating ligands (mostly TBTA or bathophenanthroline) are often employed in order to enhance the activity of the catalyst and to protect the Cu(I) from oxidation [4], the Cu(I) species was generated *in situ* by the reduction of CuSO_4_ with sodium ascorbate. Furthermore, a two-phase solvent system (CH_2_Cl_2_ as a co-solvent with water) was applied in order to facilitate the dissolution of both the steroid and the catalyst system, to eliminate the need for ligands and to simplify the reaction protocol [21].

In all cases, full conversion of the starting compound **2** or **3** was observed after overnight stirring at ambient temperature. As expected, the reactions occurred in a regioselective manner, and the triazole products (**4a**–**l** and **5a**–**l**) could generally be obtained in yields of 80–94% after chromatographic purification; there were two exceptions: **4i** and **5i,** in yields of 75% and 78%, respectively.

The structures of the newly synthetized compounds (**4a**–**l** and **5a**–**l**) were confirmed by NMR measurements. The ^1^H NMR spectra of **4a**–**i** and **5a**–**i** revealed the appearance of the new signals of the incorporated aryl groups at 6.8–8.2 ppm as compared with the spectra of the starting materials (**2** and **3**), while the 5′-H singlet was identified at 7.7–8.0 ppm. Furthermore, the aliphatic region in the spectra of **4j**–**l** and **5j**–**l** containing a cycloalkyl group was enriched by the signals of the appropriate CH_2_ and CH protons, and the singlet of 5′-H appeared at ∼ 7.3 ppm. As concerns the 16-H and 17-H signals, significant differences were observed between the two epimers. For **4a**–**l** the multiplet of 16-H was identified at ∼ 5.4 ppm, while the signal of 17-H appeared as a doublet (*J* = 5.0 Hz) at 4.1–4.2 ppm, whereas in the spectra of **5a**–**l** the 16-H multiplet was found at ∼ 4.7 ppm, and the 17-H doublet (*J* = 7.0 Hz) at 4.1–4.2 ppm. The 13C NMR spectra of **4j**–**l** also contained the signals of the heteroaromatic ring, one for C-5′ at ∼ 120 ppm, and the other for C-4′ at ∼ 147 ppm.

### Determination of the antiproliferative properties of 16α-triazolylestrone derivatives

The antiproliferative properties of the prepared 16α-triazolylestrone derivatives were determined on a panel of human cancerous cell lines (HeLa, A431 and MCF7) by means of the MTT assay in a two-step procedure. Two final concentrations (10 and 30 μM) were first applied for all compounds. For agents exhibiting a growth of inhibition at least 60% against any of the cell lines, further assays with lower concentrations were performed and the IC_50_ values were calculated. The cancer selectivities of these compounds were additionally determined by the same MTT assay against the noncancerous normal lung fibroblast cell line MRC5 (Table 1). The configuration of the OH group at position 17 did not have a consequent effect on the cell growth, although the β configuration seemed to be preferred. Since azidoalcohols **2** and **3** did not exhibit substantial action, the presence of the triazole ring is considered to be essential for the effect. Derivatives with an unsubstituted Ph ring (**4a** and **5a**) and those containing simple substituents (**4b**–**d** and **5b**–**d**) or cycloalkyl groups (**4j**–**l** and **5j**–**l**) also exerted moderate action. Introduction of a carbon chain (ethyl, propyl or *tert*-butyl) on the aromatic moiety, however, resulted in increased activities, and these molecular elements combined with 17β-hydroxy groups generated the most potent members of the current set (**5f**–**h**). The *m*-aminophenyl-substituted heteroaromatic ring resulted in another effective compound, but in this case the 17α-hydroxy epimer (**4i**) proved to be more potent. On the basis of their antiproliferative effects, compounds **4i** and **5f**–**h** were selected for further experiments, including characterization of the cancer selectivity. All four steroids exerted limited action on the proliferation of noncancerous fibroblast MRC5. In the cases of **4i** and **5h,** 50% inhibition was not elicited up to 30 μM.

**Table 1:** Antiproliferative properties of the synthetized compounds.

Compound	Conc. (μM)	Inhibition % ± SEM[Calculated IC_50_ value]^a^			
		HeLa	MCF7	A431	MRC5
**2**	10	–^b^	–	29.83 ± 2.36	n.d.^c^
	30	30.24 ± 2.25	–	51.48 ± 1.87	
**3**	10	–	–	–	n.d.
	30	24.44 ± 2.51	–	47.71 ± 2.22	
**4a**	10	37.44 ± 2.44	–	34.92 ± 0.58	n.d.
	30	68.28 ± 0.54	49.93 ± 0.63	47.92 + 1.24	
		[10.21 μM]	[>30 μM]	[>30 μM]	
**4b**	10	–	–	–	n.d.
	30	–	–	–	
**4c**	10	–	–	–	n.d.
	30	–	–	–	
**4d**	10	–	–	24.68 ± 1.31	n.d.
	30	45.45 ± 0.84	28.79 ± 1.51	26.13 ± 2.30	
**4e**	10	51.34 ± 0.62	34.13 ± 2.14	41.87 ± 1.92	n.d.
	30	71.39 ±1.17	64.39 ± 1.15	55.47 ± 0.79	
		[14.80 μM]	[17.78 μM]	[22.76 μM]	
**4f**	10	47.07 ± 1.06	61.87 ± 2.59	–	n.d.
	30	97.30 ± 0.49	96.36 ± 0.44	29.21 ± 2.76	
		[10.68 μM]	[8.07 μM]	[>30 μM]	
**4g**	10	–	–	–	n.d.
	30	51.62 ± 2.04	–	23.24 ± 1.03	
**4h**	10	–	24.19 ± 2.00	23.71 ± 1.03	n.d.
	30	92.85 ± 0.41	66.63 ± 1.19	42.59 ± 1.14	
		[11.68 μM]	[11.58 μM]	[>30 μM]	
**4i**	10	47.24 ± 2.13	–	26.98 ± 0.87	–
	30	98.38 ± 0.15	82.48 ± 0.85	94.70 ± 0.46	25.74 ± 2.94
		[13.85 μM]	[14.88 μM]	[11.75 μM]	[>30 μM]
**4j**	10	23.78 ± 2.27	30.96 ± 1.71	35.79 ± 1.53	n.d.
	30	51.49 ± 1.92	43.48 ± 1.30	49.96 ± 1.43	
**4k**	10	25.33 ± 2.54	–	–	n.d.
	30	38.09 ± 2.03	47.94 ± 1.15	–	
**4l**	10	–	–	–	n.d.
	30	34.44 ± 2.14)	33.29 ± 2.51	26.92 ± 1.75	
**5a**	10	–	–	–	n.d.
	30	34.63 ± 2.14	39.95 ± 1.96	52.94 ± 0.70	
**5b**	10	40.54 ± 0.74	–	44.29 ± 0.73	n.d.
	30	50.06 ± 1.13	–	44.71 ± 1.63	
**5c**	10	–	–	–	n.d.
	30	36.57 ± 1.08	44.20 ± 1.54	58.09 ± 0.21	
**5d**	10	37.41 ± 1.16	26.05 ± 2.73	–	n.d.
	30	46.76 ± 2.95	37.10 ± 2.35	49.14 ± 2.13	
**5e**	10	31.65 ± 2.53	–	–	n.d.
	30	40.81 ± 2.35	–	–	
**5f**	10	90.47 ± 0.53	73.15 ± 1.39	72.94 ± 0.87	32.75 ± 2.49
	30	95.17 ± 0.27	78.94 ± 0.55	70.98 ± 0.86	68.32 ± 0.76
		[5.08 μM]	[7.88 μM]	[6.77 μM]	[17.64 μM]
**5g**	10	85.62 ± 0.75	44.84 ± 2.19	47.32 ± 1.02	33.74 ± 1.74
	30	95.55 ± 0.62	60.60 ± 1.97	73.60 ± 0.46	68.61 ± 1.22
		[8.69 μM]	[10.78 μM]	[10.68 μM]	[17.07 μM]
**5h**	10	75.26 ± 1.57	32.80 ± 2.59	43.15 ± 1.87	21.01 ± 1.58
	30	86.44 ± 0.57	36.30 ± 1.26	51.76 ± 1.52	20.44 ± 1.29
		[12.11 μM]	[>30 μM]	[>30 μM]	[>30 μM]
**5i**	10	–	–	–	n.d.
	30	24.55 ± 2.69	64.62 ± 1.71	–	
**5j**	10	–	–	–	n.d.
	30	40.47 ± 2.39	–	–	
**5k**	10	26.28 ± 1.23	–	–	n.d.
	30	34.55 ± 1.61	–	–	
**5l**	10	–	–	–	n.d.
	30	26.54 ± 2.16	–	–)	
**Cisplatin**	10	42.61 ± 2.33	53.03 ± 2.29	88.54 ± 0.50	72.30 ± 2.30
	30	99.93 ± 0.26	86.90 ± 1.24	90.18 ± 1.78	70.65 + 1.34
		[12.43 μM]	[9.63 μM]	[2.84 μM]	[4.51 μM]

^a^ Mean value from two independent determinations with five parallel wells, standard deviation less than 15%.

^b^ Inhibition values <20% are not presented for clarity.

^c^ n.d.: not determined.

### Cell cycle analysis of HeLa cells treated with triazole-containing estrane

**Fig 2:**
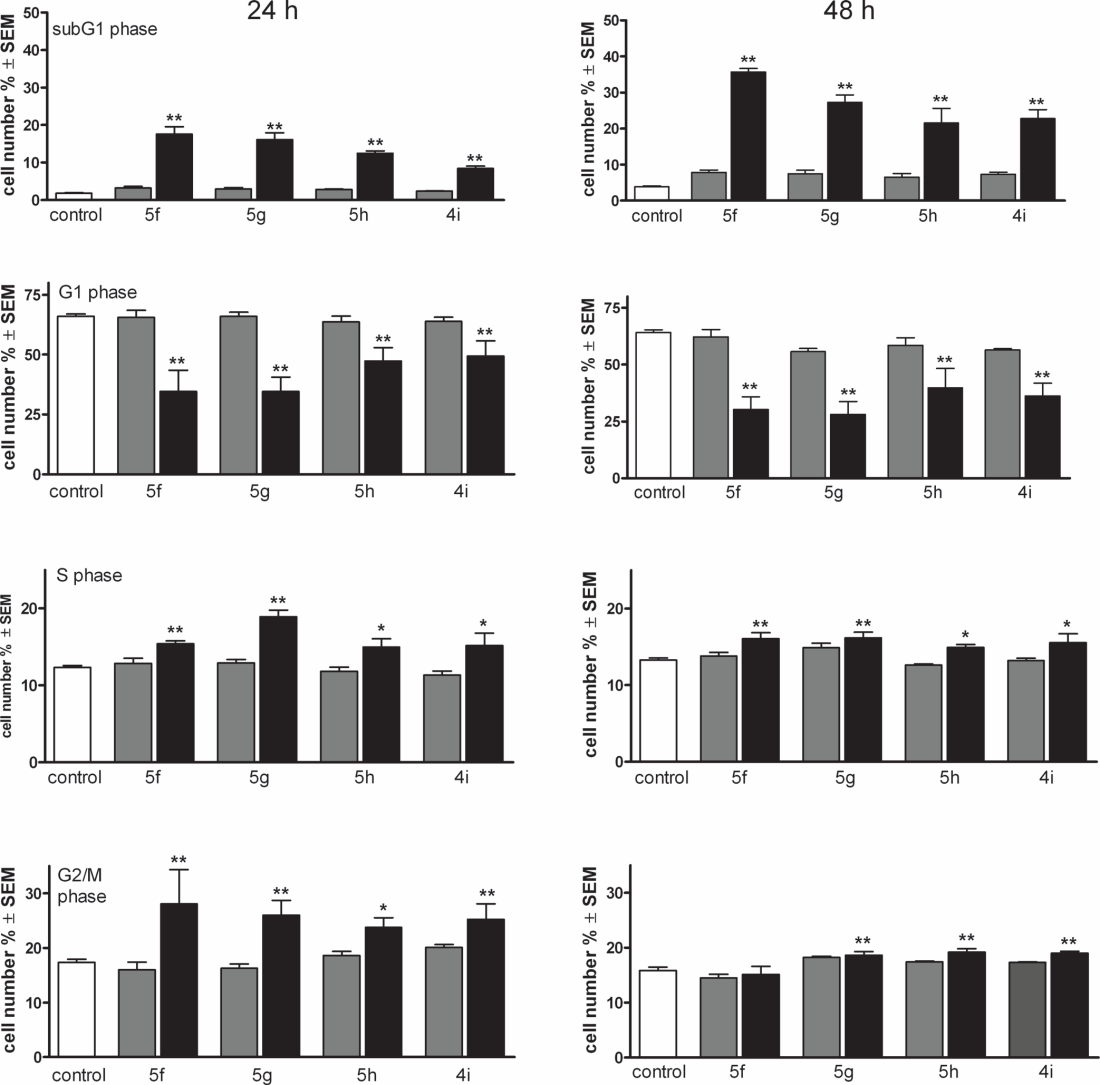
Effects of compounds 5f, 5g, 5h and 4i on the HeLa cell cycle distribution after incubation for 24 (left panels) and 48 h (right panels). Gray and black columns indicate 3 and 10 μM, respectively. * and ** denote p < 0.05 and p < 0.01, respectively, as compared with the control cells.

HeLa cells were treated with the tested compounds at 3 and 10 μM for 24 and 48 h, and the phase distribution of the treated cells was determined (Fig. 2). Treatment with the selected estrane analogs resulted in a concentration-dependent increase of subG1 phase cells, which was more pronounced after incubation for 48 h. At the same time, the G1 populations decreased substantially, while the synthetic and G2/M phases exhibited modest, but significant increases. Though compound **5f** proved to be the most potent inducer of the hypodiploid cell population, no substantial differences were evidenced in the cell cycle distributions of the treated cells.

### Double staining with Hoechst 33258 and PI

**Fig 3:**
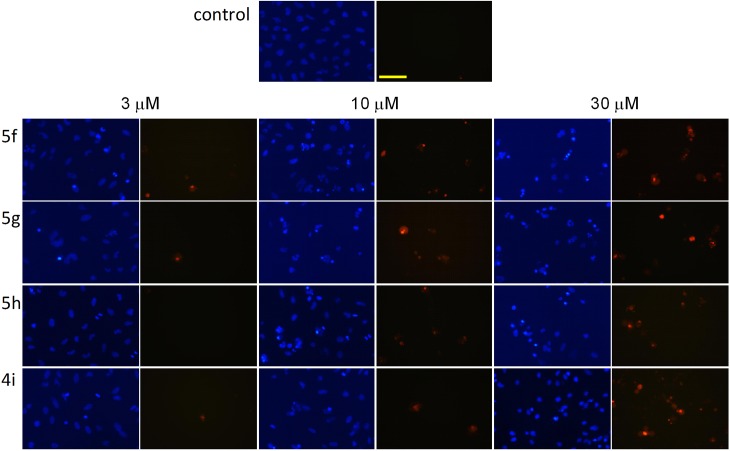
Fluorescent microscopy images of Hoechst 33258-PI double staining. Two separate pictures from the same field were taken for the two markers. HeLa cells were treated with vehicle (control), or with **5f**, **5g**, **5h** and **4i** at the indicated concentrations. The blue fluorescence (left panels) indicates Hoechst 33258, and the red fluorescence (right panels) is a consequence of PI accumulation. The bar in the PI control picture indicates 100 μm.

HeLa cells were treated with the steroids in concentrations of 3, 10 and 30 μM and then incubated for 24 h and stained with the fluorescent DNA markers Hoechst 33258 and PI in order to evaluate the morphological markers of the effects induced by the tested compounds. Two separate pictures from the same field were taken for the two fluorescent dyes. Morphological changes such as nuclear condensation, the appearance of apoptotic bodies and increase of the cell membrane permeability were recognized in a concentration-dependent manner as evidence of apoptosis and necrosis (Fig. 3). All four selected steroids (**4i**, **5f**, **5g** and **5h**) in a concentration of 3 μM induced early apoptosis, as confirmed by nuclear condensation without increased membrane permeability. The gradual impairment of the membrane function was detected by more frequent PI staining on increase of the applied concentration, which may be evidence of late apoptosis or necrosis. Treatment with 30 μM of these compounds resulted in disturbed membrane permeability, without the corresponding nuclear condensation indicating the necrosis-inducing capacity of the agents.

**Fig 4:**
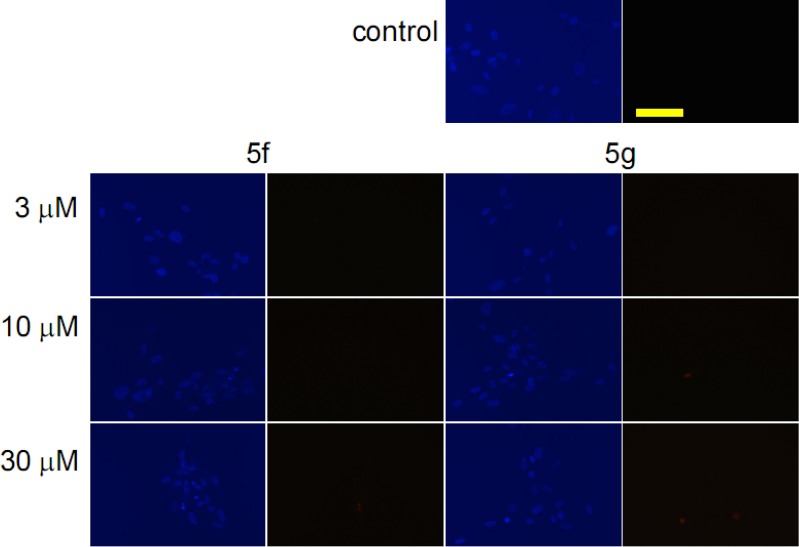
Fluorescent microscopy images of Hoechst 33258-PI double staining. Two separate pictures from the same field were taken for the two markers. MRC5 cells were treated with vehicle (control), or with **5f** and **5g** at the indicated concentrations. The blue fluorescence (left panels) indicates Hoechst 33258, and the red fluorescence (right panels) is a consequence of PI accumulation. The bar in the PI control picture indicates 100 μm.

Since two of the selected steroids (**5f** and **5g**) elicited substantial antiproliferative effects on MRC5 cells too, the staining was extended to these noncancerous cells with identical concentrations and incubation period (Fig. 4). Sparse nuclear condensation was evidenced in fibroblast cells treated with higher concentrations (10 or 30 μM), without marked PI staining.

### Caspase-3, caspase-8 and caspase-9 assays

**Fig 5:**
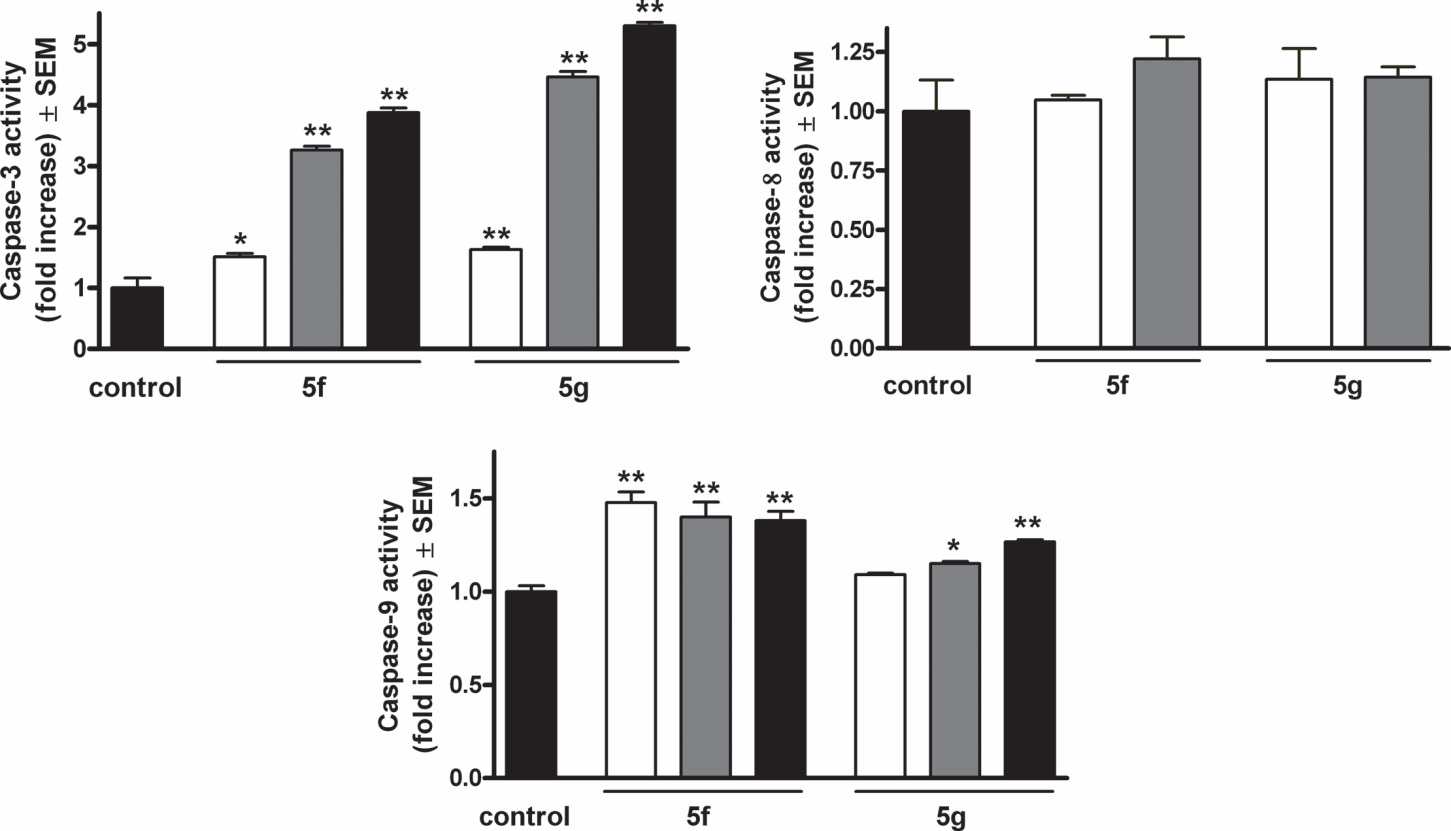
Induction of caspase-3, caspase-8 and caspase-9 activities after incubation with compounds 5f and 5g for 24 h. White, gray and black columns denote 3, 10 and 30 μM of the given agent. * and ** denote p < 0.05 and p < 0.01, respectively, as compared with the control condition.

On the basis of the results of cell cycle analysis and Hoechst 33258 and PI double staining, the effects of two selected compounds (**5f** and **5g**) on the activities of the apoptotic key enzymes caspase-3, caspase-8 and caspase-9 were determined. Both steroid analogs activated the executive caspase-3 in a concentration-dependent way during a 24-h exposure (Fig. 5). Agent **5g** exerted more pronounced action in this respect. The activity of the initiator caspase-9 was also significantly increased by both agents, though the extents were less pronounced. 24 h of exposure to agent **5f** resulted in similarly elevated enzyme activities in the concentration range 3–30 μM, while agent **5g** caused concentration-dependent caspase-9 activation. On the other hand, none of the tested agents elicited significant activation of caspase-8.

### RT-PCR studies

**Fig 6:**
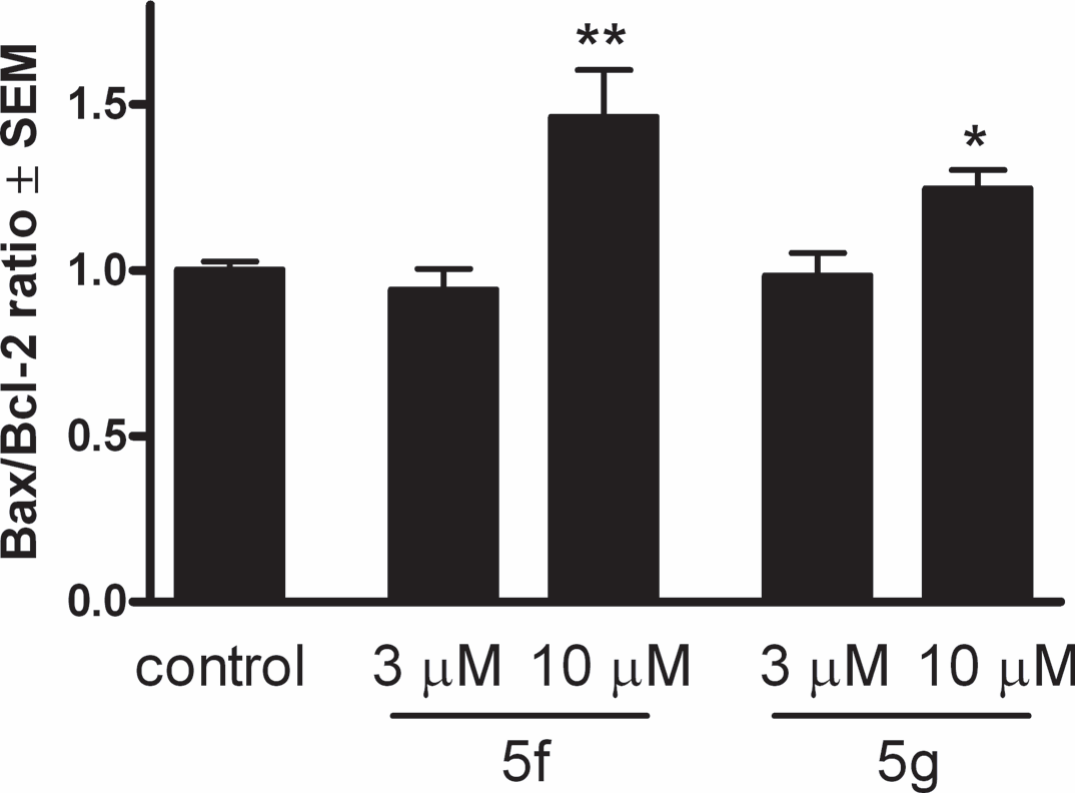
Effects of compounds 5f and 5g on the Bax/Bcl-2 ratio after incubation of HeLa cells for 24 h. * and ** denote p < 0.05 and p < 0.01, respectively, as compared with the control condition.

The expressions of the cell cycle regulator factors of the G2–M transition (CDK1, cyclin B1, cyclin B2 and cdc25B) and factors that play key roles in the mitochondrial pathway of apoptosis (Bax and Bcl-2) were determined at the mRNA level by means of RT-PCR. From the results of cell cycle analysis and caspase-3 and caspase-9 assays, the effects of the two most effective compounds at 3 and 10 μM on these mRNA sequences were determined following a 24-h incubation. Two well-characterized proteins responsible for the regulation of outer mitochondrial membrane permeability, Bax and Bcl-2, did not exhibit substantial differences (data not shown). However, under otherwise the same conditions, the ratio Bax/Bcl-2 was significantly higher at the higher concentration for both compounds (Fig. 6). This indicates activation of the intrinsic pathway of apoptosis.

**Fig 7:**
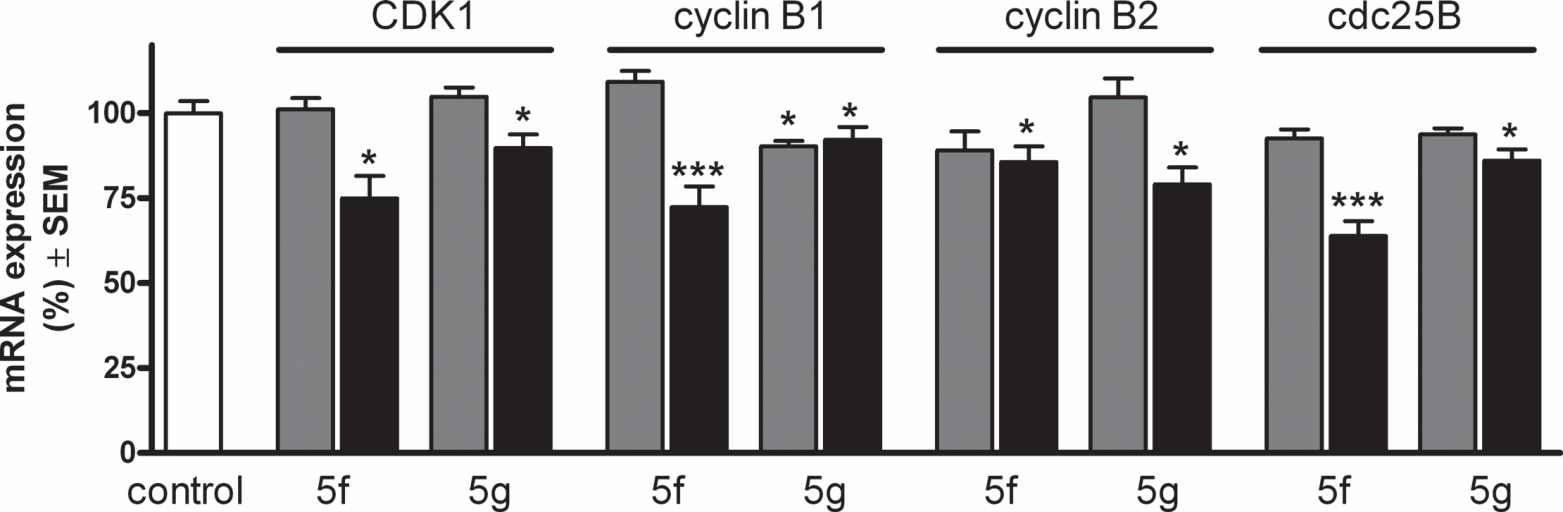
Expression of CDK1, cyclin B1, cyclin B2 and cdc25B at the mRNA level after incubation with 3 (gray columns) or 10 (black columns) μM of compounds 5f or 5g. *, ** and *** denote p < 0.05, p < 0.01 and p < 0.001, respectively, as compared with the control condition.

All four selected factors responsible for the G2–M transition were decreased after treatment with the higher concentration (10 μM). Moreover, the expression of cyclin B1 at the mRNA level was significantly reduced even after treatment with 3 μM of **5g** (Fig. 7).

### Western blotting studies

**Fig 8:**
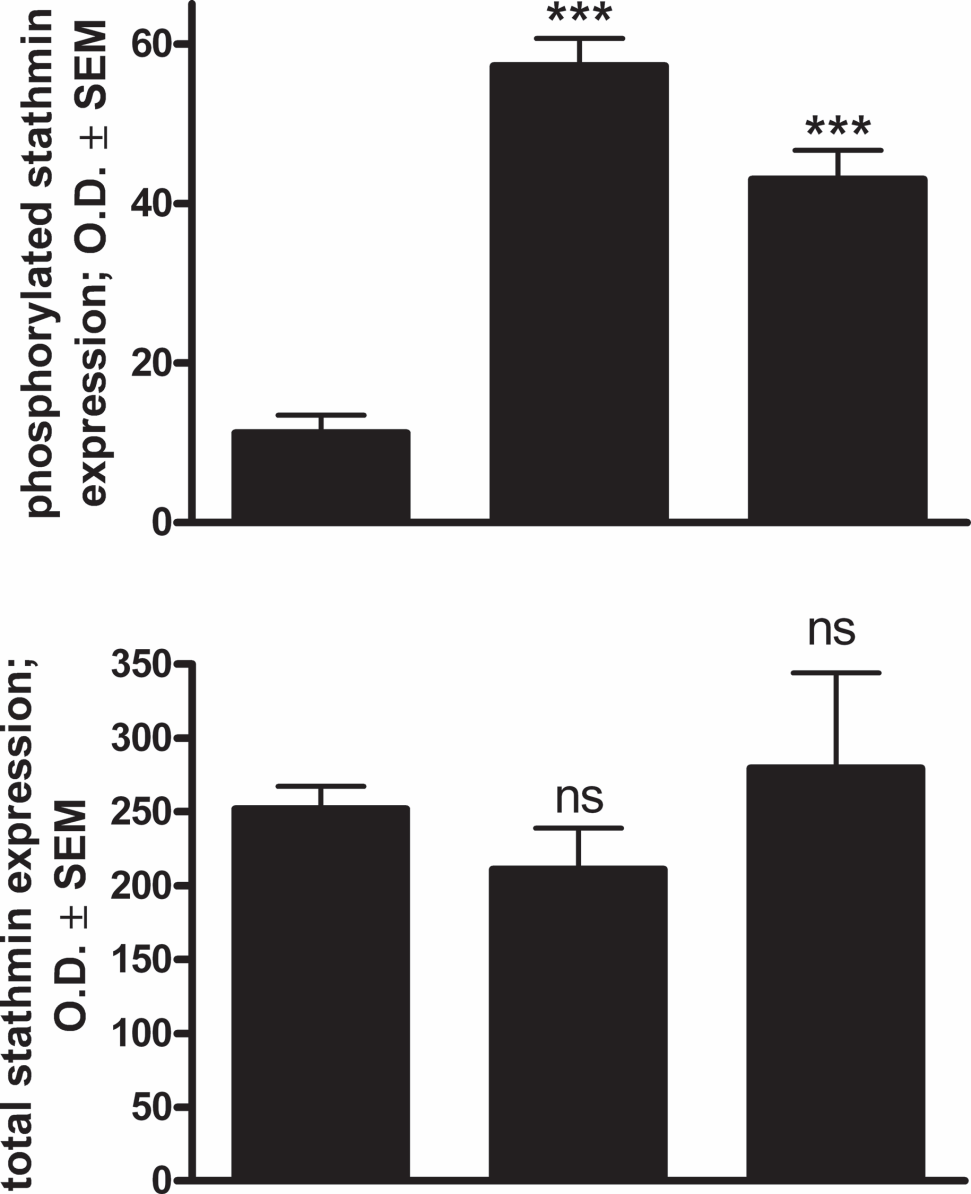
Effects of 5f and 5g (10 μM) on the expression of phosphorylated (upper panel) and total (lower panel) stathmin protein in HeLa cells after incubation for 48 h, determined by western blot analysis. Results are mean values ± SEM of the data on two separate measurements, n = 6. ns indicates p > 0.05, *** indicates p < 0.001 as compared with the untreated control cells. Panels below are representative membrane pictures.

In response to a 48-h exposure to 10 μM **5f** or **5g**, the protein expression of phosphorylated stathmin, a microtubule destabilizing protein, was significantly increased severalfold as compared to untreated control cells (Fig. 8). On the other hand, the total amount of stathmin did not display any significant alteration indicating a change in the phosphorylation state of the protein.

## Discussion

Compounds with a steroidal skeleton may exert an extremely broad variety of pharmacological activities in spite of the conserved chemical structure. The steroid backbone can therefore be utilized for the design and synthesis of a wide range of drug candidates.

A convincing amount of evidence has accumulated concerning the anticancer efficacy of natural steroids isolated from plants and their synthetic analogs. The cancer-preventing properties of cardiotonics (e.g. digoxin and digitoxin) traditionally used in the treatment of congestive heart failure have been recognized in retrospective epidemiological analyses [33]. These findings suggested the extension of the field of indication of the currently utilized digitaloids, and intensive research has therefore been initiated to develop a digitalis-based, novel class of antitumor agents [34].

The antiproliferative action of a set of solanidine analogs against HeLa cells was reported recently. Some of these steroids substantially inhibited the outflow of rhodamine-123 from murine lymphoma cells, mediated by the ABCB1 transporter [35].

Many steroidal alkaloids, including α-tomatidine and solanidine, inhibited the growth of human cancer cell lines at reasonable concentrations, whereas this action was less pronounced for their aglycones tomatidine and solanidine, respectively, indicating that the attractive properties of the steroid scaffold can be improved by an appropriately selected smaller substituent [36]. This concept was utilized in our current work, in which a substituted triazolyl ring was introduced onto the estrane core.

Estrogens are generally regarded as cellular proliferation-potentiating factors, and this is especially true for some malignancies of gynecological origin. Of the many estrane-related antiproliferative compounds recently described, the most widely investigated (and even subjected to clinical trial) is an endogenous metabolite of estrone, 2-methoxyestradiol, which exerts its anticancer effect by eliciting an imbalance of the microtubule dynamics and the direct inhibition of neoangiogenesis. [37, 38].

A set of estrone-16-oxime ethers were recently synthetized and tested for anticancer properties. The most potent analogs inhibited DNA synthesis in Hela cells, changed the expression of endogenous factors regulating the G1–S transition (retinoblastoma protein, CDK4 and p16) and induced apoptosis [39].

Since most, if not all, of the currently used anticancer agents possess the ability to initiate programmed cell death by modifying the balance between apoptotic and antiapoptotic signaling, the demonstration of apoptosis induction is a critical step in the development of an anticancer drug candidate [40]. Treatment with each of the four selected molecules (**4i**, **5f**, **5g** and **5h**) for 24 h, even at the lowest concentration (3 μM), resulted in nuclear condensation with minimal or no disruption in membrane permeability, which is a morphological marker of apoptosis. Flow cytometry was utilized for a quantitative description of the cell cycle distribution of the treated cells. The most effective agents increased the hypodiploid (subG1) population in a concentration- and time-dependent manner. The reduced DNA stainability is considered to be a consequence of the progressive loss of DNA due to activation of endonuclease and the elimination fragments as part of the self-decomposition during apoptosis [28].

Since the present compounds contain an estrane skeleton, interaction with estrogenic receptors seems a possible mechanism of action. Additionally, their potential estrogenic activity is a reasonable question. On the basis of a well-established structure–activity relationship, any action mediated through estrogenic receptors can be excluded [41]. The 3-OH group of estradiol is needed as an H-bond donor in its interaction with its receptors, and the 3-methyl ether of estradiol exhibits less than 1% relative binding affinity. A bulky 16-alpha substituent is another structural feature which abolishes the estrogenic activities of estranes.

Caspases are cysteinyl aspartate proteinases present in almost all intact cells as inactive precursors which become activated by proteolytic cleavage upon receiving apoptotic stimuli. In mammals, 18 caspases have been identified and are classified into initiator and executionary caspases according to their role in the apoptotic machinery [42]. In spite of the fact that both caspase-3 and caspase-9 have been implicated in non-apoptotic functions, the activation of these enzymes in cancer cell cultures can still be regarded as an indication of apoptotic execution and initiation of its intrinsic pathway, respectively [43]. From the aspect of the caspase activation pattern of the two selected compounds, it may be pointed out to that they induce apoptosis via the intrinsic pathway. The activation of caspase-9 seems less pronounced, which is not unusual in view of the fact that this enzyme is the first element in a cascade, while caspase-3 is a terminal element and therefore a product of amplification.

Since the mitochondria maintain the capacity to initiate the controlled cellular decomposition upon receiving appropriate signals, they serve as a central hub in the regulation of the apoptosis–survival balance. The mitochondrial pathway of apoptosis is induced by the permeabilization of its outer membrane, resulting in cytochrome c release and the subsequent formation of the apoptosome, a multiprotein complex acting as a scaffold for successive events of apoptosis. The permeabilization of the outer mitochondrial membrane is therefore a crucial event and strictly regulated by the members of the Bcl-2 protein family. Although an antiapoptotic subfamily, including Bcl-2, Bcl-xl and Mcl-1, maintains a balance with some proapoptotic proteins (Bax and Bak), Bcl-2 and Bax are frequently investigated representative members of the two subgroups [44]. Their relation is generally regarded as a marker of the apoptotic–survival balance [45]. The higher concentrations of both selected compounds (**5f** and **5g**) resulted in a significant increase in the ratio Bax/Bcl-2, reinforcing the mitochondrial origin of the detected apoptosis.

Cell division is a highly complex procedure involving an incompletely described array of regulatory steps, most of which are controlled by reversible protein phosphorylation. Specifically, the activity of the cyclin B–CDK1 complex is pivotal in regulating the G2–M phase transition, and especially in the initiation of chromosome condensation [46]. CDK1 is maintained inactive during most of the cell cycle through phosphorylation by Wee1 and Myt1 kinases. When CDK1 activity becomes required for the progression into the M phase, cdc25 phosphatase dephosphorylates the CDK1-containing complex. In mammals, three isoforms of cdc25 have been identified: cdc25A, cdc25B and cdc25C. The overexpression of CDC25A and CDC25B is reported to be involved in carcinogenesis and is associated with poor prognosis [47]. The functional differences between these isoforms have not been fully elucidated, but the normal development of CDC25B and CDC25C double knockout mice indicates that CDC25A is capable of performing all the essential operations [48]. CDC25B was proposed to be responsible for the initial activation of cyclin B–CDK1 complex during G2–M phase transition in HeLa cells [49]. This led us to select the most terminal executive regulators of cell cycle transition for determination of their expression at the mRNA level. Since the activity of the cyclin B–CDK1 complex is regulated in part by phosphorylation, and CDC25B is also phosphorylated by the complex itself, the mRNA level expression appears to be inadequate for a complete description of the tested steroids on the regulatory network. In spite of the limitations of the applied PCR technique, it seems relevant that the expressions of all four regulating factors were significantly decreased, indicating that the intervention in the cell cycle machinery is likely to occur at upstream levels.

Stathmin or oncoprotein 18 is a highly conserved oncoprotein frequently overexpressed in cancer cells which plays a crucial role in the early phase of mitosis, destabilizing the microtubules [50]. It is regulated by phosphorylation on four serine residues after turning off its destabilizing activity, and the cell can enter mitosis. Upon increased phosphorylation of stathmin, therefore, the accumulation of cells in the G2/M phase could be expected. This consideration is in agreement with the presented results and published findings. Treatment with an innovative formulation of paclitaxel resulted in a pronounced increase of gastric cancer cells in the G2/M phase and also the increased phosphorylation of stathmin [51]. Since this phosphorylation can be effected by a broad set of kinases, including CDK1 and CDK2, calmodulin-dependent protein kinase, and cAMP and cGMP dependent protein kinases, the exact enzyme responsible for the action of the presented steroids remains unclear.

Cancer selectivity is one of the most crucial parameters determining the decision as to the further development of a drug candidate. A viability assay and fluorescent staining on intact human fibroblast cells can certainly not be regarded as a complete toxicological evaluation. However, it is clearly promising that two of the four selected molecules (**5f** and **5g**) exhibited higher calculated IC_50_ values against noncancerous than against malignant cells, and did not elicit substantial membrane damage up to 30 μM. A further one of the selected agents (**4i**) did not lead to 50% inhibition of fibroblast growth up to 30 μM, and the fourth (**5h**) proved selective for HeLa cells.

## Conclusions

A set of estrone derivatives containing a substituted 16α-triazolyl on ring D were synthetized by click chemistry and investigated for their antiproliferative action on human adherent cell lines. Some of them exhibited *in vitro* potencies comparable to that of the clinically utilized reference agent cisplatin. The most potent analogs were subjected to additional investigations in order to characterize their pharmacological properties. Activation of the intrinsic pathway of apoptosis was evidenced by biochemical and morphological markers. Cell cycle blockade at the G2–M transition was additionally proved. The presented data demonstrate that estrone may be regarded as a suitable skeleton for the design of innovative antiproliferative drug candidates.

## Supporting Information

S1 Fig Growth rates of utilized cell lines. (DOCX)

S2 Fig NMR spectra of the most effective compounds (4i, 5f, 5g and 5h). (DOCX)

S1 Table Primers and PCR conditions of the determined genes, the Genebank access numbers and the length of PCR products. (DOCX)
