# Supplementary material for: A Click Approach to Novel D-Ring-Substituted 16α-Triazolylestrone Derivatives and Characterization of Their Antiproliferative Properties
Source: PLoS One. 2015 Feb 18;10(2):e0118104. doi: 10.1371/journal.pone.0118104 (PMC4333823; doi:10.1371/journal.pone.0118104)

**Figure S1:** Growth rates of utilized cell lines

For each cell line, 500,000 cells were seeded into a culture flask (day 1) and the total cell numbers were determined in the next three days. Average fold increases were calculated from triplicate determinations by means of a Z1 Coulter Counter (Beckman Coulter, Brea, CA, USA).


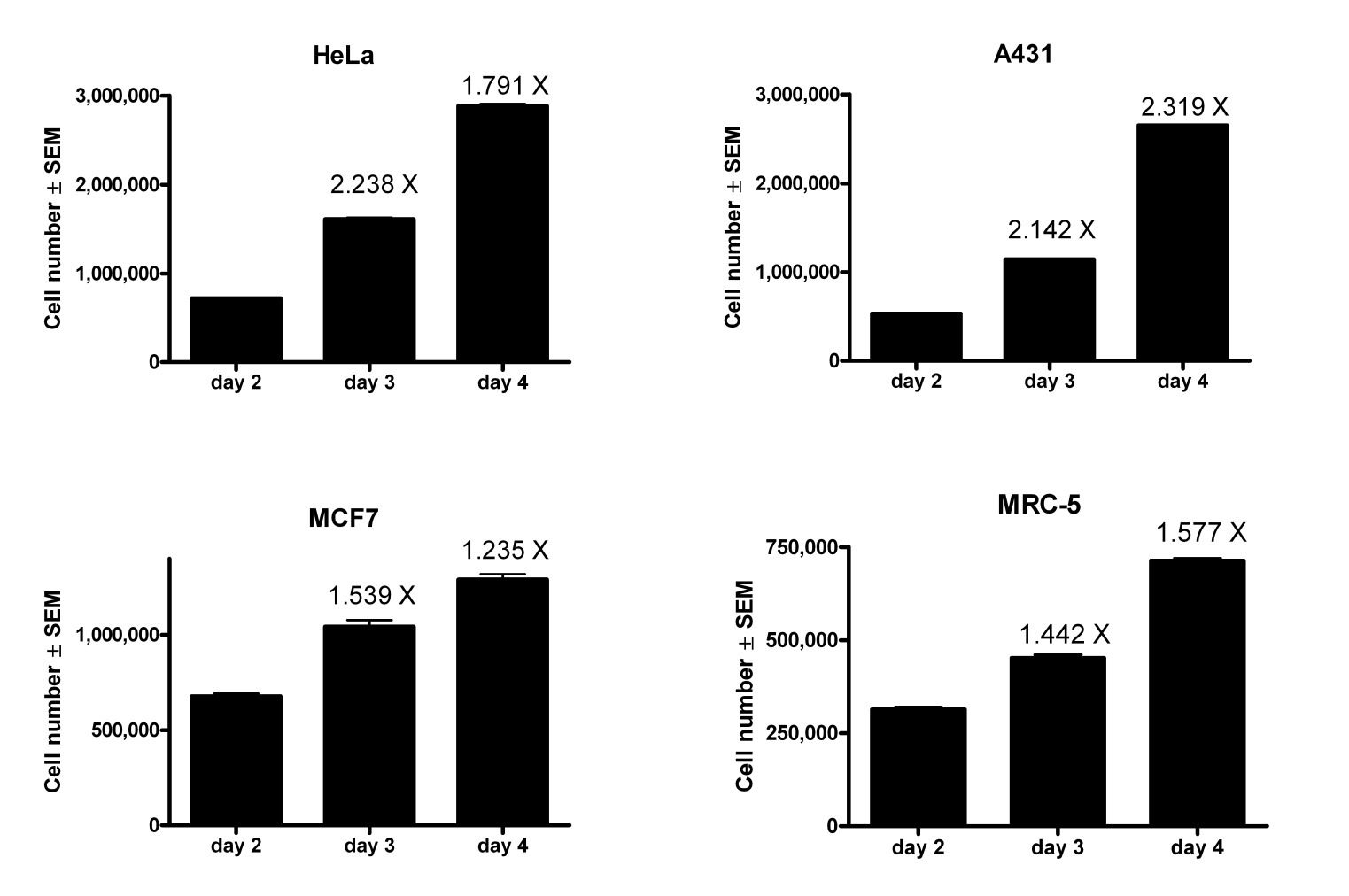

Supplement: S1 Fig — (DOCX) [file pone.0118104.s001.docx]
