# Supplementary material for: A Click Approach to Novel D-Ring-Substituted 16α-Triazolylestrone Derivatives and Characterization of Their Antiproliferative Properties
Source: PLoS One. 2015 Feb 18;10(2):e0118104. doi: 10.1371/journal.pone.0118104 (PMC4333823; doi:10.1371/journal.pone.0118104)

**Figure S2:** NMR spectra of the most effective compounds (4i, 5f, 5g and 5h)

NMR spectra of compound **4i** in CDCl_3_:


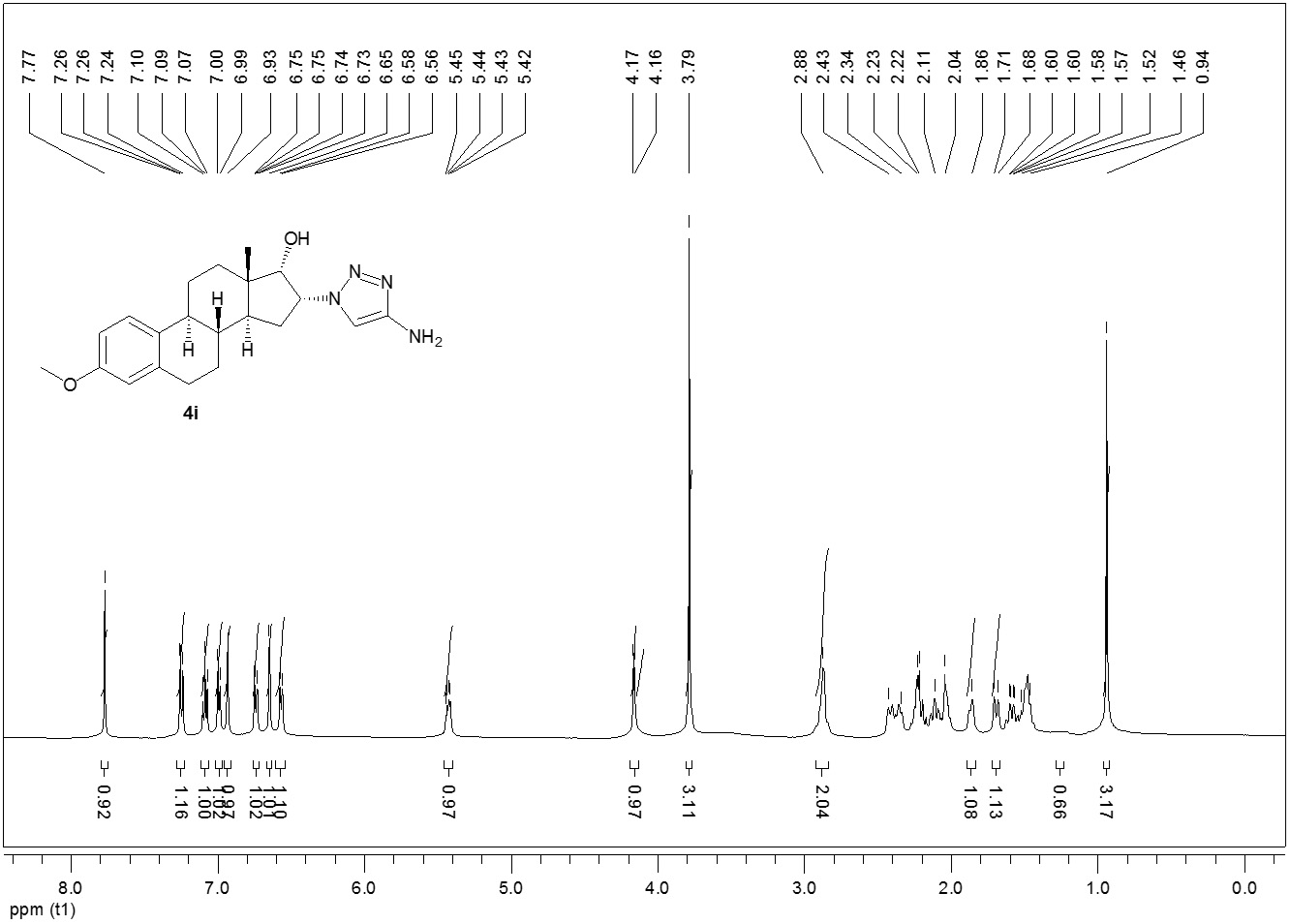


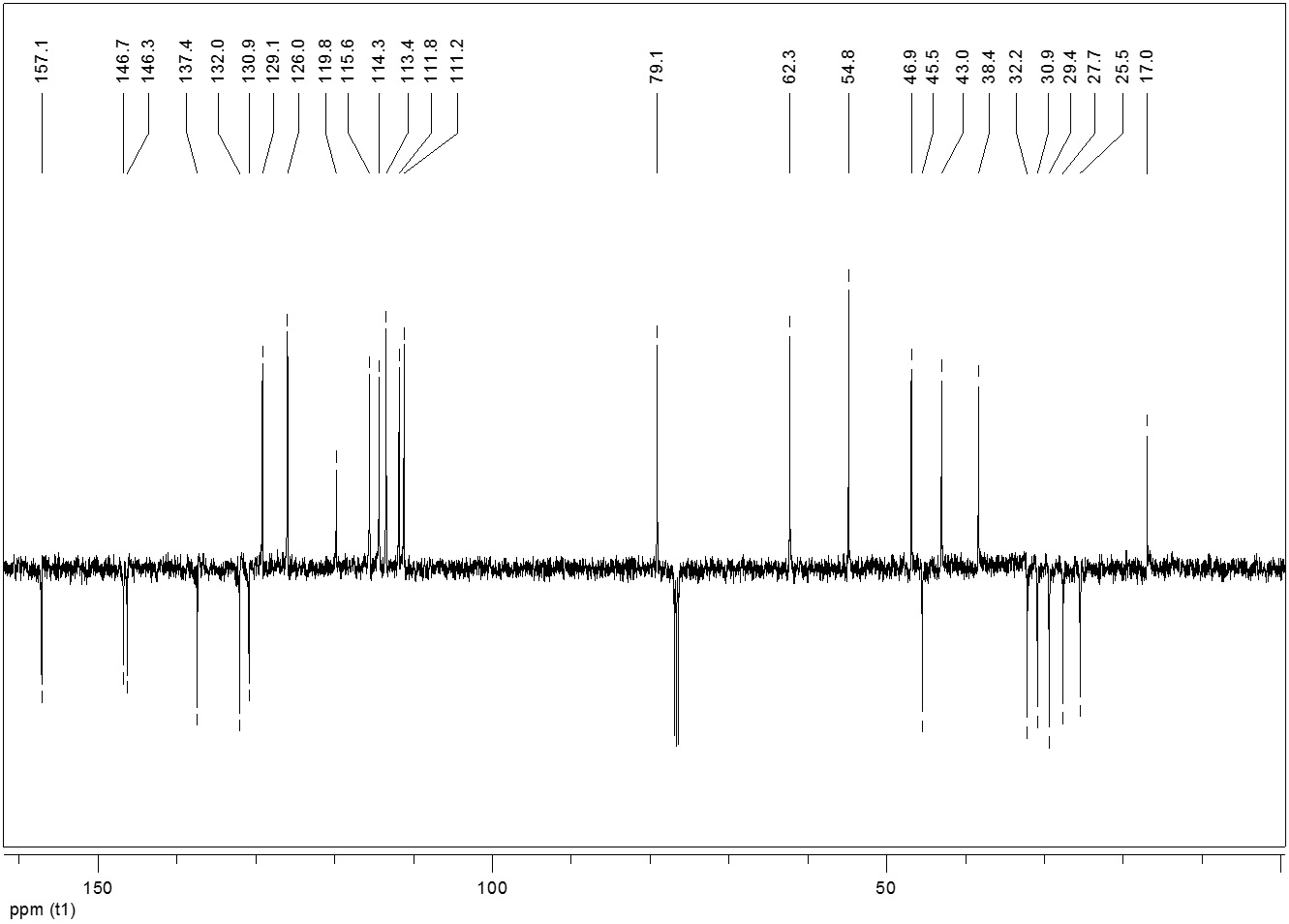


^1^H-NMR spectra of compound **5f** in CDCl_3_:


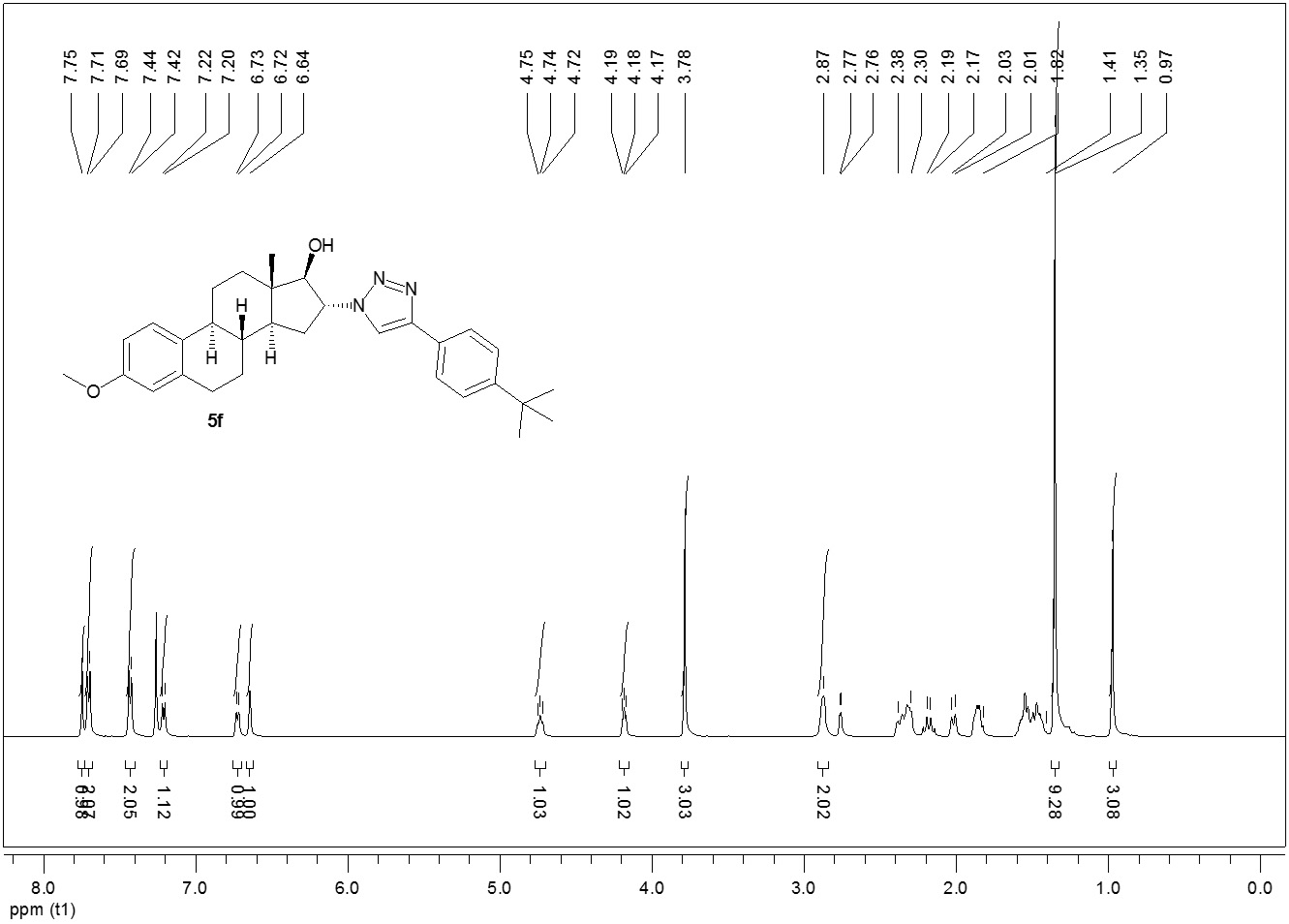


^13^C-NMR spectra of compound **5f** in DMSO-*d*_6_


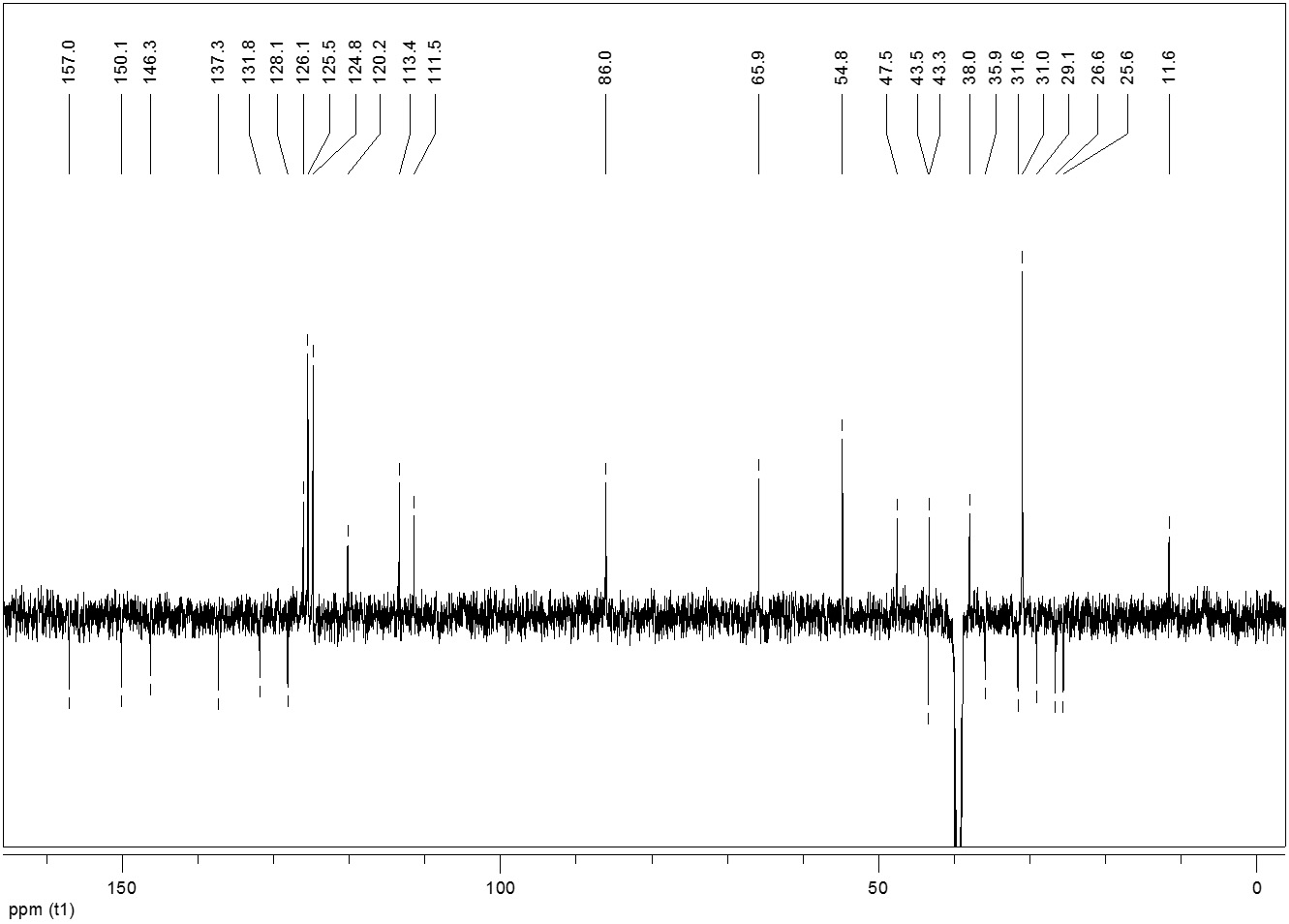


NMR spectra of compound **5g** in DMSO-*d*_6_:


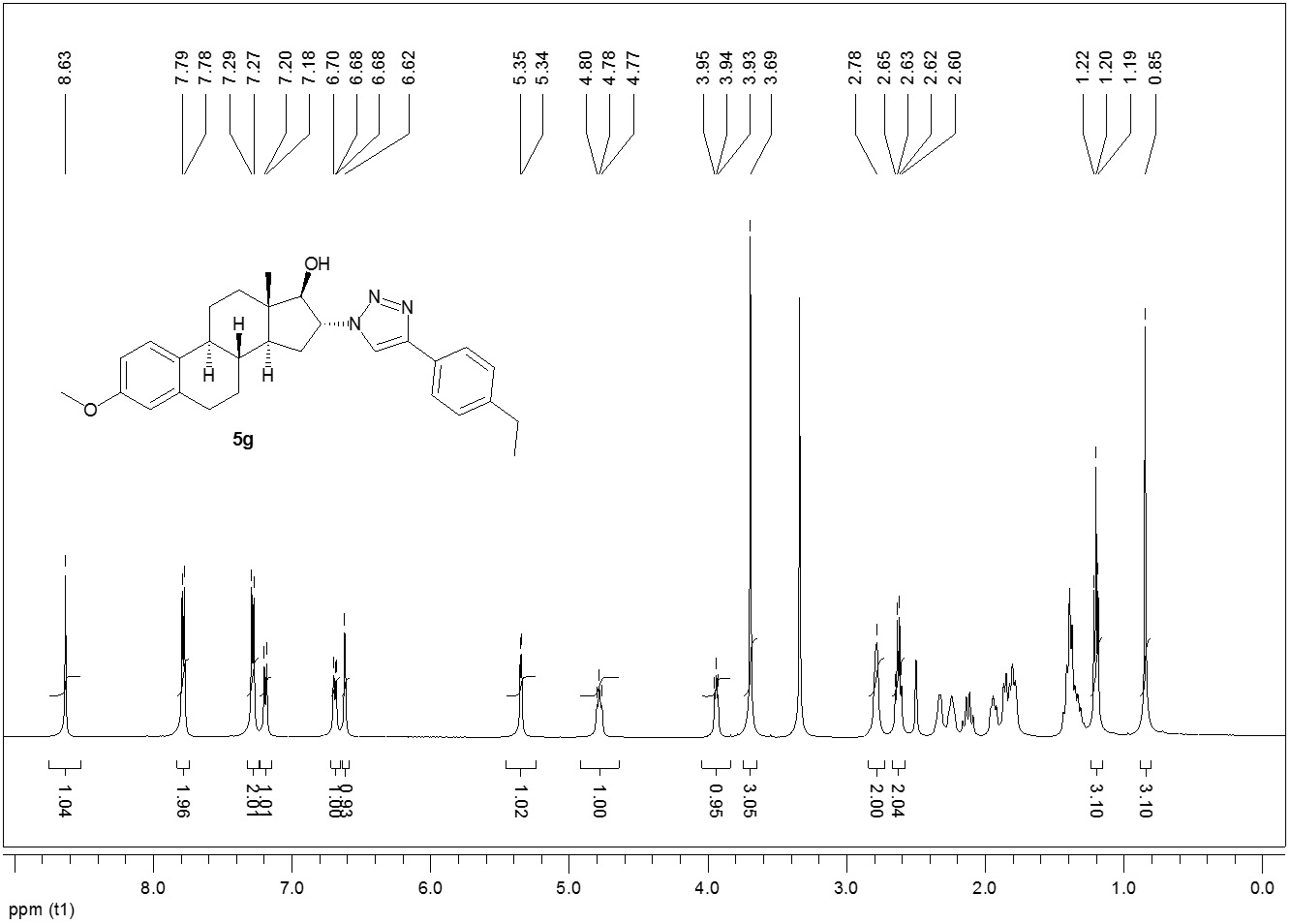


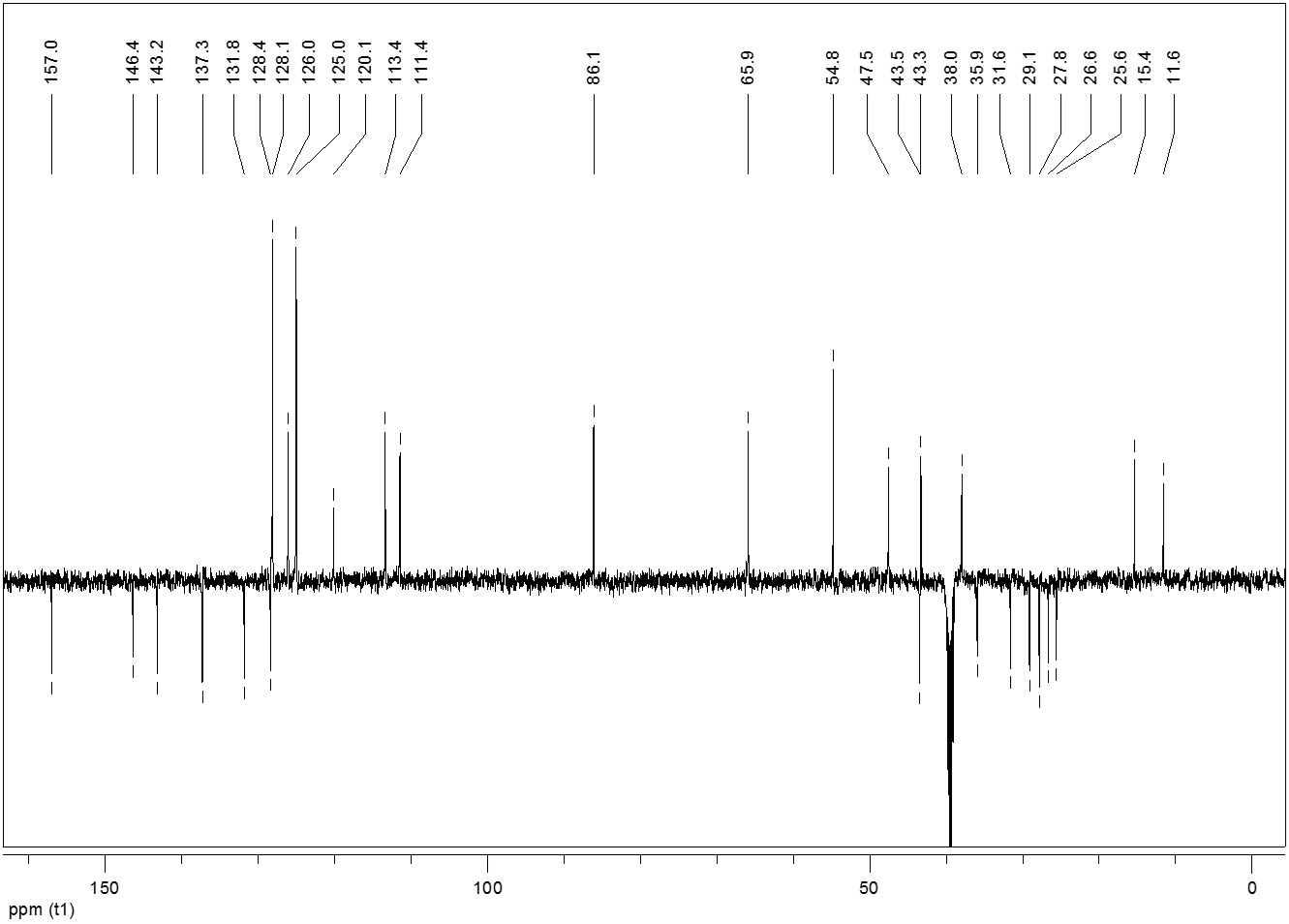


NMR spectra of compound **5h** in DMSO-*d*_6_:


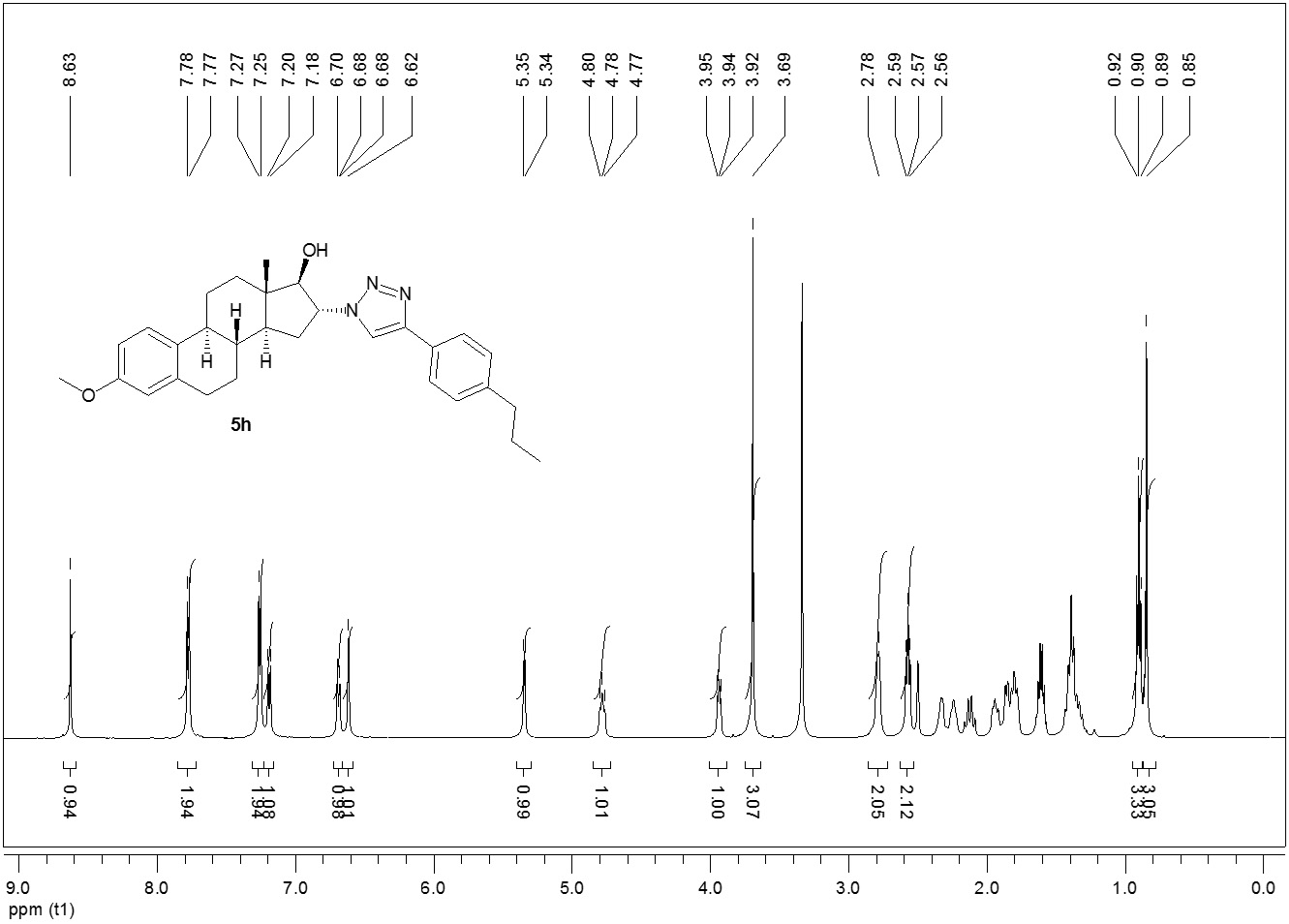


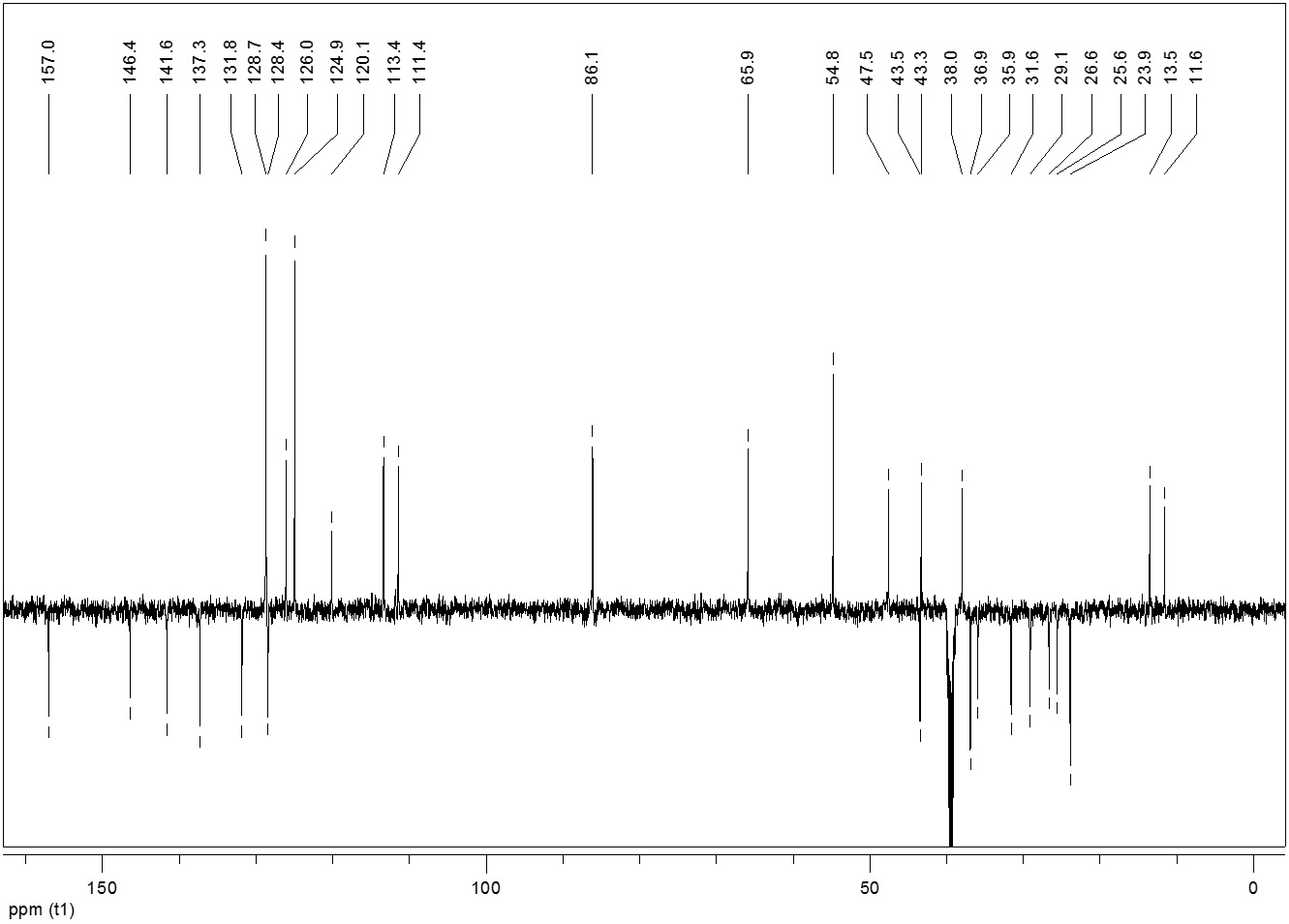

Supplement: S2 Fig — (DOCX) [file pone.0118104.s002.docx]
