# Supplementary material for: A Click Approach to Novel D-Ring-Substituted 16α-Triazolylestrone Derivatives and Characterization of Their Antiproliferative Properties
Source: PLoS One. 2015 Feb 18;10(2):e0118104. doi: 10.1371/journal.pone.0118104 (PMC4333823; doi:10.1371/journal.pone.0118104)
Supplement: S1 Table — (DOCX) [file pone.0118104.s003.docx]

**Table** **S1:** Primers and PCR conditions of the determined genes, the Genebank access numbers and the length of PCR products

| **Name:** | **Primer sequence** | **Gene ID** | **Product size**  **(bp)** | **Coupling temp.**  **(°C)** | **Cycle number** |
| --- | --- | --- | --- | --- | --- |
| CDK1 | F: ACTGGCTGATTTTGGCCTTGCC  R: TGAGTAACGAGCTGACCCCAGCAA | 983 | 118 | 62 | 34 |
| cyclin B1 | F: AATAAGGAGGGAGCAGTGCG  R: GAAGAGCCAGCCTAGCCTCAG | 891 | 51 | 60 | 36 |
| cyclin B2 | F: GCGTTGGCATTATGGATCG  R: TCTTCCGGGAAACTGGCTG | 9133 | 51 | 60 | 35 |
| Cdc25B | F: CACGCCCGTGCAGAATAAGC  R: ATGACTCTCTTGTCCAGGCTACAGG | 994 | 417 | 60 | 34 |
| Bax | F: TGGCAGCTGACATGTTTTCTGAC  R: CGTCCCAACCACCCTGGTCT | 581 | 195 | 53 | 29 |
| Bcl-2 | F: GACTTCGCCGAGATGTCCAG  R: CAGGTGCCGGTTCAGGTACT | 596 | 225 | 51 | 29 |
| hGAPDH | F: ACCCAGAAGACTGTGGATGG  R: TGCTGTAGCCAAATTCGTTG | 2597 | 415 | 55 | 24 |
